# Genome-wide meta-analysis of 158,000 individuals of European ancestry identifies three loci associated with chronic back pain

**DOI:** 10.1371/journal.pgen.1007601

**Published:** 2018-09-27

**Authors:** Pradeep Suri, Melody R. Palmer, Yakov A. Tsepilov, Maxim B. Freidin, Cindy G. Boer, Michelle S. Yau, Daniel S. Evans, Andrea Gelemanovic, Traci M. Bartz, Maria Nethander, Liubov Arbeeva, Lennart Karssen, Tuhina Neogi, Archie Campbell, Dan Mellstrom, Claes Ohlsson, Lynn M. Marshall, Eric Orwoll, Andre Uitterlinden, Jerome I. Rotter, Gordan Lauc, Bruce M. Psaty, Magnus K. Karlsson, Nancy E. Lane, Gail P. Jarvik, Ozren Polasek, Marc Hochberg, Joanne M. Jordan, Joyce B. J. Van Meurs, Rebecca Jackson, Carrie M. Nielson, Braxton D. Mitchell, Blair H. Smith, Caroline Hayward, Nicholas L. Smith, Yurii S. Aulchenko, Frances M. K. Williams

**Affiliations:** 1 Seattle Epidemiologic Research and Information Center (ERIC), Department of Veterans Affairs Office of Research and Development, Seattle, Washington, United States of America; 2 Division of Rehabilitation Care Services, VA Puget Sound Health Care System, Seattle, Washington, United States of America; 3 Department of Rehabilitation Medicine, University of Washington, Seattle, Washington, United States of America; 4 Medical Genetics, Department of Medicine, University of Washington, Seattle, Washington, United States of America; 5 Polyomica, ‘s-Hertogenbosch, the Netherlands; 6 Laboratory of Theoretical and Applied Functional Genomics, Novosibirsk State University, Novosibirsk, Russia; 7 Laboratory of Recombination and Segregation Analysis, Institute of Cytology and Genetics SD RAS, Novosibirsk, Russia; 8 Department of Twin Research and Genetic Epidemiology, King’s College London, London, United Kingdom; 9 Department of Internal Medicine, Erasmus Medical Center, Rotterdam, the Netherlands; 10 Institute for Aging Research, Hebrew SeniorLife, Boston, Massachusetts, United States of America; 11 Department of Medicine, Beth Israel Deaconess Medical Center and Harvard Medical School, Boston, Massachusetts, United States of America; 12 California Pacific Medical Center Research Institute, San Francisco, California, United States of America; 13 Department of Public Health, University of Split Medical School, Split, Croatia; 14 Cardiovascular Health Research Unit and Department of Medicine, University of Washington, Seattle, Washington, United States of America; 15 Department of Biostatistics, University of Washington, Seattle, Washington, United States of America; 16 Department of Medicine, University of Göteborg, Göteborg, Sweden; 17 Department of Medicine, School of Medicine, University of North Carolina, Chapel Hill, North Carolina, United States of America; 18 Clinical Epidemiology Unit, Department of Medicine, Boston University School of Medicine, Boston, Massachusetts, United States of America; 19 Centre for Genomic and Experimental Medicine, MRC Institute of Genetics & Molecular Medicine, University of Edinburgh, Edinburgh, United Kingdom; 20 Geriatric Medicine, Department of Internal Medicine and Clinical Nutrition, Institute of Medicine, University of Gothenburg, Sweden; 21 Department of Internal Medicine and Clinical Nutrition, Institute of Medicine, University of Gothenburg, Göteborg, Sweden; 22 Department of Orthopedics and Rehabilitation, Oregon Health and Science University, Portland, Oregon, United States of America; 23 Department of Medicine, Oregon Health and Science University, Portland, Oregon, United States of America; 24 Institute for Translational Genomics and Population Sciences, Los Angeles Biomedical Research Institute, Harbor-UCLA Medical Center, Torrance, California, United States of America; 25 Division of Genomic Outcomes, Departments of Pediatrics and Medicine, Harbor-UCLA Medical Center, Torrance, California, United States of America; 26 Genos Ltd, Osijek, Croatia; 27 Faculty of Pharmacy and Biochemistry, University of Zagreb, Zagreb, Croatia; 28 Department of Health Services, University of Washington, Seattle, Washington, United States of America; 29 Department of Epidemiology, University of Washington, Seattle, Washington, United States of America; 30 Kaiser Permanente Washington Health Research Institute, Kaiser Permanente Washington, Seattle, United States of America; 31 Department of Orthopedics, Skane University Hospital, Lund University, Malmö, Sweden; 32 Departments of Medicine and Rheumatology, University of California Davis, Sacramento, California, United States of America; 33 Department of Genome Sciences, University of Washington, Seattle, Washington, United States of America; 34 Hospital “Sveti Ivan”, Zagreb, Croatia; 35 Departments of Medicine and Epidemiology, University of Maryland, Baltimore, Maryland, United States of America; 36 Department of Medicine, The Ohio State University, Columbus, Ohio, United States of America; 37 School of Public Health, Oregon Health and Science University, Portland, Oregon, United States of America; 38 Geriatric Research, Education and Clinical Center, Veterans Affairs Medical Center, Baltimore, Maryland, United States of America; 39 Division of Population Health Sciences, School of Medicine, University of Dundee, Dundee, United Kingdom; 40 MRC Human Genetics Unit, MRC Institute of Genetics & Molecular Medicine, University of Edinburgh, United Kingdom; Icahn School of Medicine at Mount Sinai, UNITED STATES

## Abstract

Back pain is the #1 cause of years lived with disability worldwide, yet surprisingly little is known regarding the biology underlying this symptom. We conducted a genome-wide association study (GWAS) meta-analysis of chronic back pain (CBP). Adults of European ancestry were included from 15 cohorts in the Cohorts for Heart and Aging Research in Genomic Epidemiology (CHARGE) consortium, and from the UK Biobank interim data release. CBP cases were defined as those reporting back pain present for ≥3–6 months; non-cases were included as comparisons (“controls”). Each cohort conducted genotyping using commercially available arrays followed by imputation. GWAS used logistic regression models with additive genetic effects, adjusting for age, sex, study-specific covariates, and population substructure. The threshold for genome-wide significance in the fixed-effect inverse-variance weighted meta-analysis was p<5×10^−8^. Suggestive (p<5×10^−7^) and genome-wide significant (p<5×10^−8^) variants were carried forward for replication or further investigation in the remaining UK Biobank participants not included in the discovery sample. The discovery sample comprised 158,025 individuals, including 29,531 CBP cases. A genome-wide significant association was found for the intronic variant rs12310519 in *SOX5* (OR 1.08, p = 7.2×10^−10^). This was subsequently replicated in 283,752 UK Biobank participants not included in the discovery sample, including 50,915 cases (OR 1.06, *p* = 5.3×10^−11^), and exceeded genome-wide significance in joint meta-analysis (OR 1.07, *p* = 4.5×10^−19^). We found suggestive associations at three other loci in the discovery sample, two of which exceeded genome-wide significance in joint meta-analysis: an intergenic variant, rs7833174, located between *CCDC26* and *GSDMC* (OR 1.05, p = 4.4×10^−13^), and an intronic variant, rs4384683, in *DCC* (OR 0.97, p = 2.4×10^−10^). In this first reported meta-analysis of GWAS for CBP, we identified and replicated a genetic locus associated with CBP (*SOX5)*. We also identified 2 other loci that reached genome-wide significance in a 2-stage joint meta-analysis (*CCDC26*/*GSDMC* and *DCC)*.

## Author summary

Back pain is the #1 cause of years lived with disability worldwide and one of the most common reasons for health care visits in developed countries, yet surprisingly little is known regarding the biology underlying this symptom. Chronic back pain is the major driver of the societal burden of back pain. Identifying biological pathways involved in chronic back pain through genetic association studies might reveal insights into the underlying mechanisms involved or suggest potential avenues for the development of new treatments. We conducted the first genome-wide association study meta-analysis to examine genetic variants associated with chronic back pain. We identified variants associated with chronic back pain in 158,025 individuals of European ancestry from 16 cohorts in Europe and North America, and replicated our findings in 283,752 UK Biobank participants of European ancestry not included in the discovery sample. Our study identifies three novel genome-wide significant associations with chronic back pain, and suggests possible shared genetic mechanisms with other traits such as cartilage, osteoarthritis, lumbar disc degeneration, depression, and height/vertebral development.

## Introduction

Back pain causes more years lived with disability than any other health condition worldwide.[1] Although most adults experience a new (‘acute’) episode of back pain at some point in their lives, the societal burden of back pain is driven by the minority of individuals who fail to recover from such episodes and go on to develop persistent (‘chronic’) back pain.[2] Chronic back pain (CBP) has a number of definitions but is most often considered as back pain of duration ≥3 months in clinical practice, and a duration of ≥6 months is also commonly used in research.[3, 4]

Back pain is moderately heritable. Meta-analysis of 11 twin studies of back pain indicates a heritability of 40%, with a pattern of monozygotic (*r*_MZ_ = 0.56) and dizygotic (*r*_DZ_ = 0.28) twin correlations suggesting an additive genetic model (2*r*_DZ_ = *r*_MZ_).[5, 6] Heritability is greater for chronic than for acute back pain.[7] Nevertheless, genetic studies attempting to identify specific genetic markers for CBP have to date been limited to small studies using the candidate gene approach.[8, 9] Although CBP is often attributed to anatomic changes such as intervertebral disc degeneration or disc herniation, such findings have only weak association with CBP, [10, 11] and explain only a small proportion (7–23%) of the genetic influence on back pain[12]. The unexplained genetic contribution to CBP may involve not only spine pathology but also functional predisposition to chronic pain involving higher-order neurologic processes related to the generation and maintenance of pain.[13–15] Furthermore, psychological factors such as depression are widely recognized as important risk factors for CBP.[16] Given the range of processes that might contribute to CBP, the agnostic genome-wide association approach may identify novel genetic variants associated with CBP and provide insights into underlying biological mechanisms not previously considered.

This research was an international collaboration between investigators from the Cohorts for Heart and Aging Research in Genomic Epidemiology (CHARGE) Consortium Musculoskeletal Workgroup[17] and the European Union FP7 project Pain-OMICS (‘Multi-dimensional omics approach to stratification of patients with low back pain’). We conducted a meta-analysis of GWAS of CBP in adults of European ancestry from 16 community- and population-based cohorts, including those from the CHARGE and PainOmics consortia, and the UK Biobank. The aim was to identify novel associations between specific genetic markers and CBP, and elucidate the biological mechanisms underlying this condition.

## Results

### Study overview

Genome-wide discovery meta-analysis was comprised of adults of European ancestry from 16 cohorts (n = 158,025 including 29,531 CBP cases; Table 1), including 15 CHARGE cohorts and participants from the UK Biobank (UKB) interim data release (UKB1). After quality control, the number of SNPs included in the meta-analysis ranged from 6,205,227 to 9,775,703, depending on the cohort (S1 and S2 Tables). Linkage disequilibrium (LD) score regression (LDsr) was used distinguish polygenicity from potential confounding,[18] using LD scores from European ancestry 1000 Genomes data. The genome-wide significance level was defined as *p*<5×10^−8^, and suggestive significance level was defined as *p*<5×10^−7^, after using the LDsr intercept as a correction factor. For those SNPs of genome-wide suggestive significance in the discovery phase, replication was conducted in a sample of UKB European ancestry participants (UKB2) who were not part of the interim data release (n = 283,752 subjects, including 50,915 CBP cases), and a joint (discovery-replication) meta-analysis was performed. We then conducted functional characterization of variants and loci achieving genome-wide significance in the joint meta-analysis.

**Table 1:** Cohorts in meta-analysis of genome-wide association studies of chronic back pain (European ancestry).

Cohort	Study setting	Country	Sample size	Chronic back pain definition	Prevalence (%)		Age (yr)	BMI (kg/m^2^)	Women (%)
Cardiovascular Health Study (CHS)	Community	USA	2849	≥1 month of back pain in consecutive years	14.2%	Cases (n = 404)	72.1 ± 5.1	27.3 ± 5.0	73.3%
						Controls (n = 2445)	72.1 ± 5.2	26.1 ± 4.3	59.6%
Framingham Heart Study[19]	Community	USA	2673	≥6 months of back pain	21.0%	Cases (n = 561)	67.7 ± 9.3	28.8 ± 5.8	62.2%
						Controls (n = 2112)	66.4 ± 9.1	28.0 ± 52	52.9%
Generation Scotland	Population	UK	5071	≥3 months of back pain	26.0%	Cases (n = 1322)	54.9 ± 11.8	28.1 ± 5.7	66.7%
						Controls (n = 3749)	52.4 ± 12.7	26.4 ± 4.6	55.4%
Johnston County Osteoarthritis Project (JoCo)	Population	USA	480	≥6 months of back pain	38.8%	Cases (n = 186)	72.0 ± 8.0	31.4 ± 6.3	65.0%
						Controls (n = 294)	73.0 ± 8.0	29.3 ± 5.2	58.4%
Mr. Os Sweden									
Gothenburg	Population	Sweden	920	≥6 months of back pain	14.2%	Cases (n = 131)	75.3 ± 3.2	26.7 ± 3.9	0%
						Controls (n = 789)	75.3 ± 3.2	26.1 ± 3.4	0%
Malmo	Population	Sweden	948	≥6 months of back pain	10.8%	Cases (n = 102)	75.8 ± 3.1	27.2 ± 3.7	0%
						Controls (n = 846)	75.6 ± 3.2	26.4 ± 3.6	0%
Mr. Os US	Community	USA	4615	≥6 months of back pain	14.1%	Cases (n = 653)	74.6 ± 6.1	28.0 ± 4.1	0%
						Controls (n = 3962)	73.9 ± 5.9	27.3 ± 3.8	0%
Osteoarthritis Initiative (OAI)	Community	USA	2474	≥1 month of back pain in consecutive years	13.5%	Cases (n = 335)	61.0 ± 9.1	28.9 ± 4.7	57.9%
						Controls (n = 2139)	61.7 ± 9.1	28.1 ± 4.5	53.2%
Rotterdam Study (RS)									
RS-1	Community	Netherlands	5965	≥6 months of back pain	14.7%	Cases (n = 877)	69.1 ± 9.2	26.7 ± 4.0	72.0%
						Controls (n = 5088)	70.0 ± 9.4	26.2 ± 3.7	58.3%
RS-2	Community	Netherlands	1566	≥6 months of back pain	36.7%	Cases (n = 574)	65.3 ± 8.0	27.7 ± 4.2	66.7%
						Controls (n = 992)	64.6 ± 8.0	27.3 ± 4.1	55.4%
RS-3	Community	Netherlands	3019	≥6 months of back pain	38.2%	Cases (n = 1154)	57.4 ± 6.9	28.1 ± 4.8	59.5%
						Controls (n = 1865)	56.9 ± 6.7	27.4 ± 4.5	54.3%
Study of Osteoporotic Fractures (SOF)	Community	USA	3615	≥6 months of back pain	16.3%	Cases (n = 589)	72.1 ± 5.6	27.6 ± 5.3	100%
						Controls (n = 3026)	71.4 ± 5.2	26.6 ± 4.4	100%
10,001 Dalmatians									
Vis	Population	Croatia	251	≥3 months of back pain	22.3%	Cases (n = 56)	67.3 ± 13.2	27.8 ± 4.4	69.6%
						Controls (n = 195)	63.7 ± 12.1	26.7 ± 4.0	52.8%
Korcula	Population	Croatia	773	≥3 months of back pain	21.2%	Cases (n = 164)	64.3 ± 12.9	28.0 ± 4.3	70.7%
						Controls (n = 609)	58.0 ± 14.9	27.0 ± 41	64.0%
UK Biobank	Population	United Kingdom	120,024	≥3 months of back pain	18.0%	Cases (n = 21,600)	57.3 ± 7.9	28.5 ± 5.4	54.0%
						Controls (n = 98,424)	57.0 ± 7.9	27.4 ± 4.8	52.4%
TwinsUK	Population-based twin registry	United Kingdom	2782	≥3 months of back pain	29.6%	Cases (n = 823)	56.7 ± 12.6	27.4 ± 5.3	90.3%
						Controls (n = 1959)	54.3 ± 13.9	26.0 ± 4.9	90.0%
**Total of all cohorts**	-	-	158,025	-	-	Cases (n = 29,531)	-	-	-
						Controls (n = 128,494)	-	-	-

### Meta-analysis of GWAS of CBP

**Fig 1:**
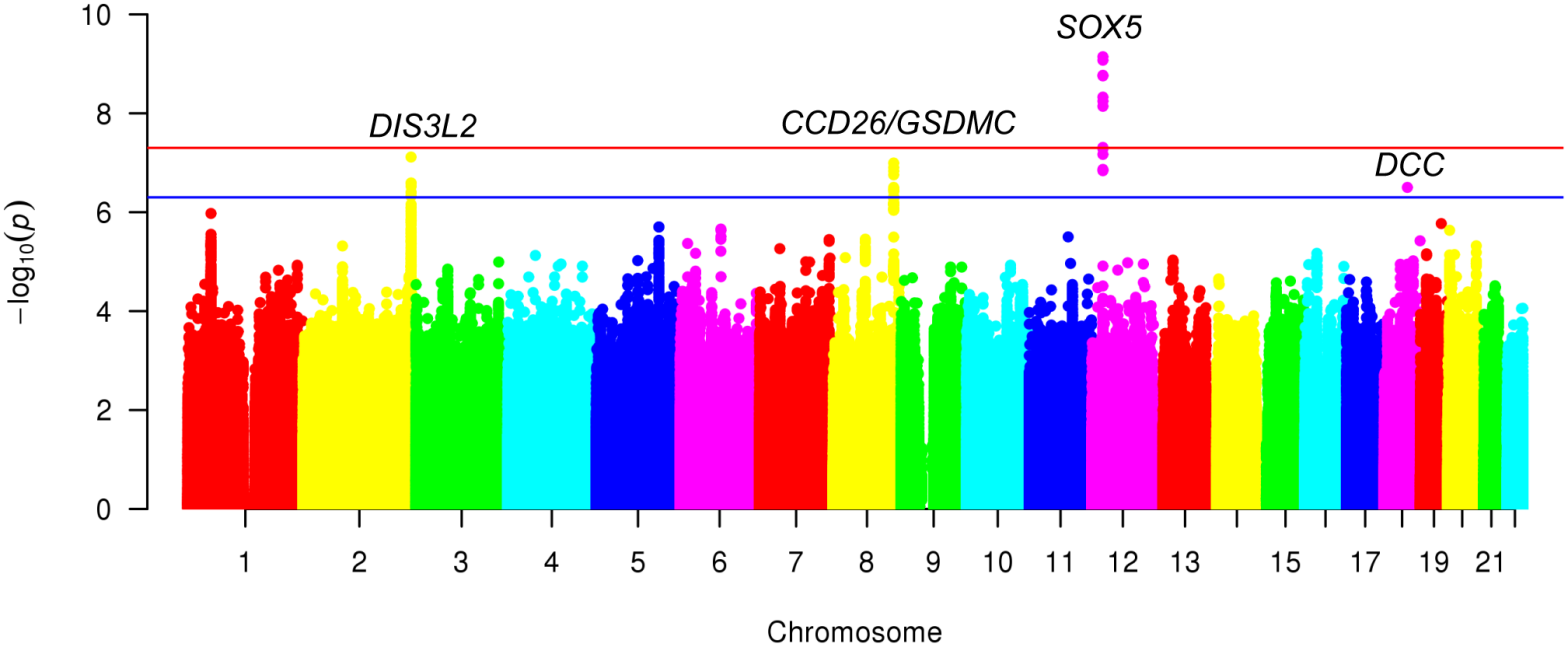
Manhattan plot for meta-analysis (discovery) of GWAS of chronic back pain (n = 158,025). GWAS = genome-wide association study. Results use the linkage disequilibrium score regression (LDSR) intercept as a correction factor. Red line depicts genome-wide statistical significance (P <5×10^−8^). Blue line depicts suggestive significance (P <5×10^−7^).

The characteristics of cohorts included in the discovery meta-analysis are shown in Table 1. The mean age of participants in each cohort ranged between 53–76 years. Within cohorts, mean age, BMI, and proportion of females was more often higher among CBP cases than among those without CBP. A quantile-quantile plot comparing the meta-analysis association results with those expected by chance is presented in S1 Fig. The LDsr intercept was 1.007 (standard error 0.006), λ was 1.114, and the LDsr ratio was 0.0581 (standard error 0.053), providing no evidence of inflation of p-values from population stratification. Meta-analysis results are summarized in the Manhattan plot shown in Fig 1.

A genome-wide significant association (OR 1.08, *p* = 7.2×10^−10^) was found for rs12310519 on chromosome 12 in an intronic region of *SOX5*, with little evidence for heterogeneity (*I*^2^ = 0, *p* = 0.95) (Table 2, S2 Fig). Several other signals were in high LD (*r*^2^>0.8) with the top signal (S3 Fig), but none were independently associated with CBP in analyses conditional on rs12310519.

**Table 2:** Association results for chronic back pain: Meta-analysis (discovery), replication, and joint meta-analysis*.

					Discovery (Meta-analysis of CHARGE and PainOmics cohorts + UKB1)^a^ (n = 158,025)						Replication (UKB2)^b^ (n = 283,752)			Joint Meta-Analysis (Discovery-Replication)^c^ (n = 441,777)		
SNP rsID	Chr:Pos^d^	Nearest Gene	Location	Alleles	EAF	OR	SE	p-value	I^2^	Het. p-value	OR	SE	p-value^b^	OR	SE	p-value
rs12310519^e^	12:23975219	*SOX5*	intronic	T/C	0.16	1.08	0.013	7.2 x 10^−10^	0	0.95	1.06	0.009	5.3 x 10^−11^	1.07	0.008	4.5 x 10^−19^
rs1453867	2:232917899	*DIS3L2*	intronic	T/C	0.65	0.95	0.010	7.7 x 10^−8^	13	0.31	0.98	0.007	0.021	0.97	0.006	3.9 x 10^−7^
rs7833174	8:130718772	*CCDC26/GSDMC*	intergenic	T/C	0.77	1.06	0.011	1.0 x 10^−7^	0	0.71	1.04	0.008	3.7 x 10^−7^	1.05	0.007	4.4 x 10^−13^
rs4384683	18:50379032	*DCC*	intronic	A/G	0.54	0.95	0.009	3.2 x 10^−7^	0	0.86	0.97	0.007	4.2 x 10^−5^	0.97	0.006	2.4 x 10^−10^

CHARGE = Cohorts for Heart and Aging Research in Genomic Epidemiology, UKB1 = UK Biobank participants from the interim data release[20], UKB2 = UK Biobank participants not included in the interim data release, chr:pos = chromosome:position, alleles = effect/other, EAF = effect allele frequency OR = odds ratio, het. = heterogeneity

*Top variant at each locus meeting suggestive or genome-wide significance level in discovery stage (p<5.0x10^-7^).

^a^After genomic control using the LD score regression intercept

^b^Replication for rs12310519. The threshold for significance in replication of rs12310519 was p<0.05 (0.05/1)

^c^The threshold for genome-wide significance in joint analysis was p<5×10^−8^

^d^Build GRCh37/hg19

^e^rs115392701 has merged into rs12310519

No other variants achieved genome-wide significance, but variants in three other loci reached suggestive significance (Table 2, S3 Table, S4–S9 Figs): rs1453867 (OR 0.95, *p* = 7.7×10^−8^), located in an intronic region of chromosome 2 within *DIS3L2*; rs7833174 (OR 1.06, *p* = 1.0×10^−7^), located in an intergenic region on chromosome 8 between *CCDC26* (a long non-coding RNA) and *GSDMC*; and rs4384683 (OR 0.95, *p* = 3.2×10^−7^), located in an intronic region of chromosome 18 within *DCC*. In each of these 3 regions, there was no other variant reaching the suggestive significance level in analyses conditional on the lead SNP in the region. *Post hoc* secondary analyses of the discovery sample showed effects of similar magnitude and direction between the CHARGE cohorts and the UKB interim data release for associations between the lead variants in the top 4 loci and CBP (S4 Table).

We examined these 4 top variants in 283,752 UKB individuals not included in the discovery sample (UKB2), including 50,915 cases (Table 2). For all 4 variants, the direction of association was the same in discovery and replication. The association for rs12310519 in *SOX5* replicated in UKB2 (OR 1.06, *p* = 5.3×10^−11^), and exceeded genome-wide significance in the joint analysis (OR = 1.07, *p* = 4.5×10^−19^). Of the 3 suggestive-significance variants from the discovery stage, rs7833174 at *CCDC26/GSDMC* (OR 1.05, *p* = 4.4×10^−13^) and rs4384683 in *DCC* (OR 0.97, *p* = 2.4×10^−10^) exceeded genome-wide significance in the joint meta-analysis, but rs1453867 in *DISL32* (OR 0.98, *p* = 3.9×10^−7^) did not (Table 2). Thus, we demonstrate genome-wide significant associations of CBP with loci tagged by rs12310519 (*SOX5)*, rs7833174 (*CCDC26/GSDMC)*, and rs4384683 (*DCC*), with replication for rs12310519 in *SOX5*.

### Characterization of variants in SOX5, CCDC26/GSDMC, and DCC

Functional characterization followed the same steps for each of the 3 loci that achieved genome-wide significance in the joint meta-analysis. First, we examined cross-phenotype genetic associations between each lead SNP and traits with possible conceptual links to CBP, in look-ups of publicly and privately available GWAS datasets. Where the lead SNP was not present in a dataset, we examined associations with the variant in highest LD with the lead SNP. Second, we annotated lead variants and those in LD (r^2^≥0.6) for consequences on gene functions (using the combined annotation dependent depletion [CADD] score [21]), potential regulatory functions (using RegulomeDB score[22]), and effects on gene expression (using GTExv6 [23, 24]), and examined whether these variants resided in enhancer regions for selected tissues with connections to the spine or pain processing (using data from the Roadmap Epigenomics Consortium [25, 26]) (Methods, S1 Text).

#### SOX5

**Fig 2:**
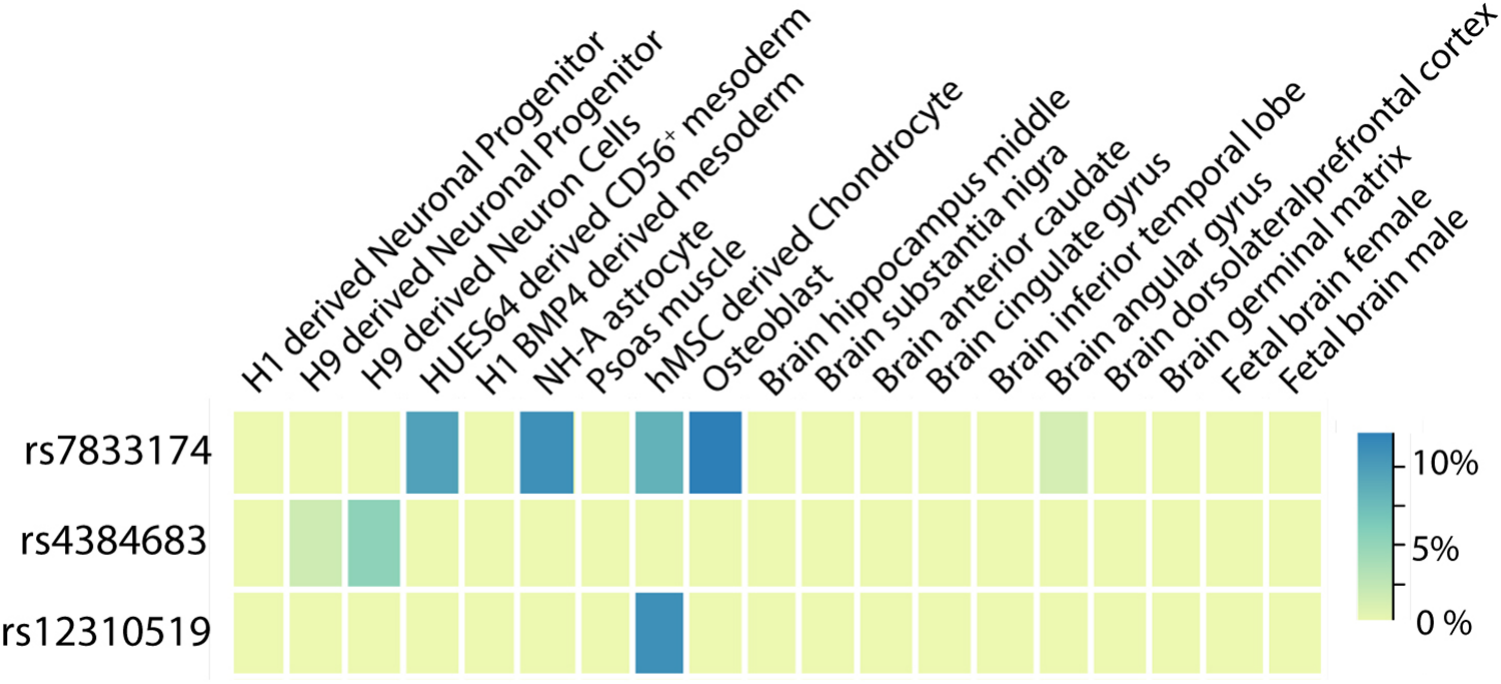
Significant variants are co-localized with potential gene regulatory markers. The heatmap depicts the percentage of variants in gene regulatory regions (associated with enhancers/promotors) in LD (r^2^>0.6) with rs7833174 (*CCDC26/GSDMC*), rs12310519 (*SOX5*), and rs4384683 (*DCC*). We examined epigenetic histone marks in selected cell types/tissues including chondrogenic, bone-related, neuronal and brain cells/tissues; mesodermal cells (related to notochord); and psoas muscle (located proximal to the lumbar spine).

Among CBP-related traits examined, the lead SNP in *SOX5*, rs12310519, was most strongly associated with imaging-detected lumbar intervertebral disc degeneration (*p* = 1.1×10^−4^; S5 Table)[27]. The highest CADD score among *SOX5* variants was 10.52 for the lead SNP in the region rs12310519, indicating it is predicted to be among the 10% most deleterious possible substitutions in the human genome (S1 Appendix). However, the overall regulatory potential of these variants was low according to RegulomeDB score (scores of 6 [‘minimal binding evidence’]) (S1 Appendix). There were no meaningful associations with gene expression using GTExv6. The lead SNP rs12310519 and variants in LD (r^2^>0.6) contained active enhancer marks in chondrogenic cells using Roadmap Epigenomics Consortium data (Fig 2, S1 Appendix).

#### CCDC26/GSDMC

In look-ups of GWAS of selected traits with possible links to CBP (S5 Table), the lead SNP rs7833174 was most strongly associated with height in UKB [28] (*p* = 1.3×10^−59^) and hip circumference in UKB [28] (*p* = 1.8×10^−5^). The lead SNP rs7833174 was also associated with radiographic hip osteoarthritis[29] (*p* = 4.9×10^−4^) (S5 Table). All variants in *CCDC26*/*GSDMC* that were suggestively associated with CBP showed cross-phenotypic associations with lumbar microdiscectomy for sciatica in a recent GWAS of Icelandic adults[30] (S6 Table, lowest *p* = 5.6×10^−12^). The direction of effect on these other phenotypes was the same as the direction of effect on CBP (i.e. the T allele associated with greater height, greater risk of radiographic hip osteoarthritis, and greater risk of lumbar discectomy for sciatica was also associated with greater CBP risk). The highest CADD score among CBP-associated variants at *CCDC26*/*GSDMC* was 18.75 for rs6470778, indicating that this SNP is predicted to be among the 5% most deleterious substitutions in the human genome, and the overall regulatory potential of these variants was substantial according to Regulome DB score (highest RegulomeDB score of 2b [‘likely to affect binding’]) (S1 Appendix). In examination of effects on gene expression using GTExv6, variants in LD with rs7833174 (r^2^>0.6) were also eQTLs for *GSDMC* expression in esophageal mucosa, skin, and skeletal muscle (S1 Appendix, *p*<5×10^−8^). The lead SNP rs7833174 and variants in LD (r^2^>0.6) contained active enhancer marks located in regulatory regions for mesodermal cells, astrocytes, chondrogenic cells, and osteoblasts in data from the Roadmap Epigenomics Consortium (Fig 2, S1 Appendix).

#### DCC

In look-ups of GWAS of selected traits with possible links to CBP (S5 Table), the lead SNP rs4384683 was associated with depressive symptoms[31](*p* = 5.9×10^−4^), with the same direction of effect (i.e the A allele was associated with less depressive symptoms and lower CBP risk). The highest CADD score among variants at *DCC* that were suggestively associated with CBP was 11.21 for rs2116378, indicating that rs2116378 is predicted to be among the 10% most deleterious substitutions in the human genome, but the overall regulatory potential of these variants was low according to Regulome DB score (highest RegulomeDB score of 5) (S1 Appendix). There were no meaningful associations with gene expression using GTExv6. In data from the Roadmap Epigenomics Consortium, the lead SNP rs4384683 and variants in LD (r^2^>0.6) contained active enhancer marks in H9 human embryonic stem cell-derived neural cells (Fig 2, S1 Appendix).

### Secondary analyses to examine interrelationships with height

*Post hoc* analyses among UKB1 participants from the discovery sample (n = 120,023) indicated that associations with CBP for the lead variant in *SOX5* were similar with and without adjustment for height as a covariate, and in conditional analyses accounting for height (S1 Text, S7 Table). Associations with CBP for the lead variant in *CCDC26*/*GSDMC* were also similar with and without adjustment for height as a covariate. However, associations with CBP were markedly diminished when conditional on the lead height-associated variant in the region, and associations with height were markedly diminished when conditioned on the lead CBP-associated variant in the region (S7 Table). This suggests that the same functional variant is responsible for association of *CCDC26*/*GSDMC* locus with height and CBP, although an alternative explanation is two functional variants in tight LD. Associations with CBP for the lead variant in *DCC* were similar with and without adjustment for height as a covariate, and in conditional analyses accounting for height (S7 Table).

To examine possible causal effects of height on CBP, we conducted a two-sample Mendelian randomization (MR) analysis using genetic variants associated with standing height in the GIANT consortium as the exposure, and the discovery phase GWAS meta-analysis of CBP as the outcome. Results of the two-sample MR using 326 SNPs and the instruments involved are available in the S2 Appendix. These instruments explained 10.1% of the variance in height, with an average SNP-height F-statistic of 78.5, indicating substantial instrument strength. ORs for CBP were 1.10 per standard deviation increase in height with inverse variance weighted (IVW) regression (p<0.0001). However, there was significant heterogeneity among SNPs (I^2^ = 0.35; p<0.0001), suggesting horizontal pleiotropy for at least some SNPs (S3 Appendix). Estimates with other MR methods were directionally consistent, and all but MR-Egger regression were statistically significant: an OR for CBP of 1.09 per standard deviation increase in height with MR-Egger regression (p = 0.19); 1.14 per standard deviation increase in height with the weighted median method (p<0.0001), and 1.17 per standard deviation increase in height with the weighted mode method (p = 0.02) (S3 Appendix). The magnitude of MR estimates after excluding 14 outlier SNPs were very similar to the two-sample MR using 326 SNPs, but with substantially less heterogeneity amongst SNPs (I^2^ = 0.12; p = 0.04), just exceeding nominal significance (S2 and S3 Appendices). MR-Egger intercepts were close to 0 with both the 326 SNP and 312 SNP instruments, and neither were statistically significant, suggesting no strong directional horizontal pleiotropy under the InSIDE (Instrument Strength Independent on Direct Effect) assumption (S3 Appendix).

#### Secondary analyses to examine for influence of relatedness in UK Biobank

GWAS analyses using linear mixed-effect models in UKB1 yielded associations between the 3 lead SNPs and CBP that were very similar to the original analyses using logistic regression in terms of statistical significance, indicating no meaningful influence on the study results due to relatedness (S8 Table).

### Heritability of CBP and genetic correlations

SNP heritability of CBP on the liability scale was 7.6%. Partitioned heritability by functional category using stratified LD score regression showed significant enrichment (p = 0.0004) for regions conserved in mammals, with 2.6% of SNPs explaining 40% of the SNP heritability of CBP, without significant enrichment for other functional categories, including coding regions (S4 Appendix). This pattern of partitioned heritability was broadly similar across cell type groups, including central nervous system (CNS), connective tissue and bone, and skeletal muscle, among others (S4 Appendix). Genetic correlations of nominal significance (range of *r*_g_ 0.17–0.31, p<0.05) were found with anthropometric traits involving obesity or body fat distribution (waist circumference, hip circumference, waist-hip ratios, overweight/obesity classes, and BMI), but not with height (S9 Table). Larger magnitude nominally significant (p<0.05) genetic correlations were also found with depression-related phenotypes (range of *r*_g_ 0.46–0.52), self-reported osteoarthritis (*r*_g_ = 0.63), and ICD-10-defined osteoarthritis (*r*_g_ = 0.49) phenotypes.

## Discussion

This study is the first meta-GWAS of CBP. This collaboration between two international consortia for genomic studies of complex traits in the USA and Europe incorporated data from 16 cohorts and more than 441,000 participants of European ancestry across discovery and replication samples. Our study identifies three novel associations with CBP for loci at *SOX5*, *CCDC26*/*GSDMC*, and *DCC*.

CBP was most strongly associated with rs12310519 in an intronic region of the *SOX5* gene. The *SOX* genes are a family of transcription factors involved in virtually all phases of embryonic development, and are thought to determine the fate of many cell types. The *SOX* genes are defined by containing the HMG (‘high mobility group’) box of a gene involved in sex determination called SRY (‘sex determining region’) [32]. *SOX5* and *SOX6* have overlapping functions and work together in close coordination that is necessary for efficient chondrogenesis [33]. Inactivation of *SOX5* leads to minor defects in cartilage and skeletogenesis in mice, whereas *SOX5/SOX6* double knockouts have severe chondrodysplasia [34]. Together with *SOX9*, *SOX5* and *SOX6* are sometimes referred to as the ‘master chondrogenic SOX trio’ [33, 35]. Prior work indicates an important role for *SOX5* in articular cartilage and osteoarthritis [36, 37], and such a role was also supported by our functional annotation showing that rs12310519 (and SNPs in high LD) overlapped with potential regulatory regions for chondrogenic cells. *SOX5/6* are also essential for notochord development, and through this role they are critical in the formation of the vertebral column, including the intervertebral discs [33, 35, 38]. Inactivation of *SOX5* and/or *SOX6* in mice leads to a range of abnormalities in the development of spinal structures [38]. Although variants in *SOX5* have not been reported in prior GWAS of limb osteoarthritis (knee, hip, or hand) [39–46], the association of *SOX5* with CBP may involve the spinal structures specifically. Consistent with this, we found a cross-phenotypic association for the lead CBP-associated variant in *SOX5* with imaging-detected lumbar intervertebral disc degeneration in a prior GWAS meta-analysis [27]. Future GWAS may also be useful to characterize other spine-related phenotypes besides disc degeneration, such as osteoarthritis of the zygapophyseal (‘facet’) joint, the only true synovial joint in the spine [47].

The intergenic variants at *CCDC26*/*GSDMC* associated with CBP in the current study were also previously found associated with lumbar microdiscectomy for sciatica due to intervertebral disc herniation.[30] These findings are intriguing, given that lumbar disc herniations (an aspect of lumbar disc degeneration) have long been implicated as a cause of some forms of back pain.[48] Recent studies have concluded that associations between imaging-detected lumbar disc herniation and CBP are of modest magnitude.[10, 11] This might explain the small magnitude association of the top variant at *CCDC26*/*GSDMC* with CBP in the current study (OR 1.08 in discovery), in contrast to the larger magnitude association seen with microdiscectomy for sciatica (OR 1.23). Functional characterization of these intergenic variants suggest the likely involvement of the gene *GSDMC*. *GSDMC* encodes the protein Gasdermin C, part of the *GSDM* family of genes that is expressed in epithelial tissues. Although the specific role of *GSDMC* in lumbar disc herniation and/or sciatica is unclear, *GSDMC* is associated with differential methylation patterns in osteoarthritis-related cartilage and subchondral bone cartilage, [49, 50] consistent with our findings that variants in LD with rs4384683 were located in potential regulatory regions in chondrocytes and osteoblasts. Our examination of univariate cross-phenotypic genetic associations for CBP-associated variants at *CCDC26*/*GSDMC* also suggest pleiotropy with radiographic hip OA for rs6470763.[29] Taken together, these data suggest interconnections between variants at *CCDC26*/*GSDMC* and CBP involving cartilage, osteoarthritis, and/or lumbar disc degeneration.

The third significant CBP-associated variant in our study was rs4384683, an intronic variant in the gene *DCC* (Deleted in Colorectal Carcinoma), which co-localized with regulatory regions in neural embryonic stem cells. *DCC* encodes a transmembrane protein that is a receptor for netrin-1, an axonal guidance molecule involved in the development of spinal and cortical commissural neurons.[51] Interactions between *DCC* and netrin-1 are among the best-studied axonal guidance processes, with key roles during development and in adulthood, and they also affect angiogenesis.[52, 53] Increased expression of netrin-1 and *DCC* occurs in degenerate human intervertebral discs compared to healthy control discs, and in nucleus pulposus compared to annulus fibrosis.[54] Netrin-1/DCC might therefore mediate neurovascular ingrowth into the intervertebral disc, which has long been implicated as a possible mechanism of chronic discogenic back pain.[54, 55] Given the well-known phenotypic correlation between depression and CBP[56], however, another possible explanation for the link between CBP and *DCC* (suggested by the cross-phenotype association of rs4384683 with depressive symptoms) is pleiotropy. Netrin-1/*DCC* interactions are also known to play a role in pain processing in the spinal cord in animal models of mechanical allodynia.[52] Taken together, this information suggests various potential mechanisms underlying the association between *DCC* and CBP, including nociceptive pathways and/or the involvement of mood.

Some epidemiological studies report that greater height confers increased risk of back pain [57–59], although a systematic review found no association.[60] Variants in *CCDC26*/*GSDMC* associated with CBP in our meta-analysis were also reported to be associated with height in prior GWAS; hence, *post hoc* analyses devoted special attention to the role of height in CBP. These region-specific analyses showed that the association of *SOX5* and *DCC* variants with CBP was independent of height; however, CBP- and height-associated variants at *CCDC26*/*GSDMC* were tightly linked and could not be disentangled in conditional analyses (S7 Table), indicating that the association of variants in *CCDC26*/*GSDMC* with both CBP and height might be explained by biological pleiotropy or mediated pleiotropy (i.e. pleiotropy due to causal effects)[61]. Mendelian randomization analysis, drawing on information from hundreds of genetic markers distributed across the genome, suggested that height may have causal effects on CBP, although with a degree of heterogeneity suggesting horizontal pleiotropy for some SNPs. Such evidence of horizontal pleiotropy is common in MR studies of complex traits,[62] and can be seen even in MR studies of exposure-outcome relationships where causal effects are known.[63] Taken together, our findings suggest a causal component to the relationship between height and CBP, but do not exclude that height and CBP are also linked by biological pleiotropy. Further more advanced studies should be conducted to corroborate our findings. Prior studies demonstrating the vital role of *SOX5* in normal vertebrate development [33, 35, 38] are a reminder that measurements of human height used for GWAS may also reflect vertebral column development; if associations with CBP and height are connected via development of the vertebral column, it will be difficult to distinguish pleiotropy and causality using genetic studies alone.

SNP-based heritability in the current study (8%) was considerably lower than estimates from twin studies (~40%). This is a common situation with modern methods of estimating heritability using genotype data, since such estimates reflect only one aspect of narrow-sense heritability captured by the additive genetic components of common variants, excluding the contributions of rare variants, non-additive effects, epistasis, or gene-environment interactions.[64] Similar to what is seen with many other human traits,[65] there was significant enrichment of SNP-based heritability of CBP for genetic regions that are conserved in mammals. Despite the modest heritability of CBP (and other self-reported traits), we found significant and large magnitude genetic correlations between CBP and other phenotypes that may be risk factors for CBP or consequences of CBP, such as depression-, osteoarthritis- and obesity-related traits (but not height). Future GWAS of CBP may benefit from taking these relationships into account, either as covariates, or in multivariate GWAS designs.

A distinguishing feature of the current study as compared to many other GWAS is that the CBP phenotype examined represents a symptom, rather than a disease or a biomarker. Although successful GWAS of self-reported symptoms have been conducted which replicate associations seen with more specific disease phenotypes,[66] our findings highlight potential challenges of GWAS of CBP: despite being one of the largest international studies of CBP ever conducted, our study detected only 3 significant associations with CBP. Still larger sample sizes will be needed in future discovery efforts using this phenotype, or different genetic approaches will be needed. A consequence of the nonspecific nature of the CBP phenotype is that, unlike other musculoskeletal phenotypes such as osteoarthritis, the tissue correlates optimal for conducting functional follow-up studies of findings from CBP GWAS are very unclear. Most animal models for back pain rely on specific mechanisms of inducing pain, such as injuries to the intervertebral disc, zygapophyseal (‘facet’) joint, dorsal root ganglion, or muscle.[67] However, each of these mechanisms likely explain only a certain portion of back pain cases, and do not encompass the important psychosocial aspects of pain and pain reporting that are highly relevant in humans. Despite the importance of psychosocial factors, our meta-GWAS findings are a reminder that structural/anatomic factors involving spinal degeneration, such as disc herniation or osteoarthritis of spinal structures (e.g. facet joints), remain potentially important contributors to the CBP. While our study accounted for age by statistical adjustment, the meta-analysis design including multiple cohorts of older adults may have led to an overrepresentation of genetic variants associated with age-related conditions, such as osteoarthritis. Future GWAS of CBP may benefit from a broader age range of participants, stratification by back pain subtypes, simultaneously studying CBP and spinal degeneration/fracture phenotypes, and examination of interactions between genetic markers for spinal degeneration and markers for pain processing or axonal signaling (including *DCC* and netrin-1).

Strengths of our study include its multicohort design and large sample size. A potential limitation of our study was heterogeneity of the CBP phenotype used, a consequence of pooling data from numerous cohorts using different definitions. Although this approach helped identify genetic associations shared across CBP subtypes, it might obscure associations pertinent to specific subtypes of back pain. As an example, we examined chronic back pain rather than chronic *low* back pain, since few of the included cohorts had available question items that isolated the low back region specifically. Given the high agreement between general back pain questions and low back-specific questions,[68] and since mid/upper back pain without concurrent low back pain is uncommon,[69] we expect that our results largely reflect genetic associations with low back pain.[70] Despite phenotype heterogeneity, which would be expected to bias the study towards the null, we successfully identified several associations of statistical significance. Recent efforts to standardize CBP definitions may help to limit phenotype heterogeneity in future meta-GWAS of CBP.[3] Another aspect of the phenotype used in our meta-analysis was that individuals with back pain of less than 3–6 months duration were included as controls. This was done deliberately so as to focus on back pain of chronic duration as the phenotype of interest. That said, GWAS examining back pain of *any* duration, or analyses excluding those with non-chronic back pain, may find different results. Another possible study limitation was lack of independence in the replication sample of UK Biobank participants from UKB2, given the same study base and methods between the UKB1 and UKB2 subcohorts. Our secondary analyses using linear mixed-effect models demonstrated similar SNP-CBP associations for our top hits when accounting for relatedness *within* UKB1, but the problem of relatedness *across* the two subcohorts (UKB1 vs. UKB2) remains. Finally, a limitation of this meta-analysis was that only autosomal variants were analysed, since some included cohorts did not analyze the X chromosome.

In summary, this meta-analysis of GWAS of CBP identified novel genetic associations with CBP at *SOX5*, *CCDC26*/*GSDMC*, and *DCC*. Analysis of data from other GWAS and functional genomics experiments suggest possible pleiotropic effects of these loci on other traits including cartilage, osteoarthritis, lumbar disc degeneration, depression, and height/vertebral development, and possible causal effects on CBP mediated through height.

## Methods

### Study design and populations

Discovery meta-analysis included adults of European ancestry from 16 population- and community-based cohorts: Cardiovascular Health Study (CHS), Framingham Heart Study (FHS), Generation Scotland (GS), Johnston County Osteoarthritis Project (JoCo), Osteoporotic Fractures in Men (MrOS) Sweden (MrOS-Gothenburg and MrOS-Malmo), MrOS US, Osteoarthritis Initiative (OAI), Rotterdam Study (RS1, RS2, and RS3), Study of Osteoporotic Fractures (SOF), 10,001 Dalmatians (Vis and Korcula), TwinsUK, and UKB participants from the interim data release[20]. Replication was conducted among UKB European ancestry participants not included in the discovery stage (UKB2), and a joint (discovery-replication) meta-analysis was performed. The separation of analyzed data from UKB into discovery (UKB1) and replication phases (UKB2) reflects the history of this scientific collaboration, in that our initial meta-analysis plan included only the UKB data then available to us and for which we had obtained approval to use (UKB1 [the interim data release]). By the time our meta-analysis was completed, all UKB data had become available; the remainder of UKB data was therefore used for replication. Detailed descriptions of the study cohorts are provided in Table 1 and the Supplemental Methods. This meta-analysis was approved by the Research and Development Committee of VA Puget Sound Health Care System (RDIS 0010, MIRB 00903). Institutional Review Board/Ethics Committee approvals at the individual study sites include those listed in the S2 Text. Written or electronic consent was provided for all studies.

### Chronic back pain (CBP) phenotype

There is no “gold-standard” definition for CBP. Consistent with the most commonly accepted clinical and research definitions for CBP [3, 4], CBP cases were defined in this study using one of 3 definitions depending on the cohort (Table 1, S10 Table): 1) ≥3 months of back pain, 2) ≥6 months of back pain, and 3) ≥1 month of back pain in consecutive years (reflecting ≥12 months of back pain). For each cohort, the comparison group (“controls”) was comprised of those who reported not having back pain or reported back pain of insufficient duration to be included as a case. This study used a general definition examining chronic ‘back pain’, as opposed to a more specific chronic ‘low back pain’ definition, due to the fact that most of the included cohorts did not include question items permitting localization of pain to the low back or lumbar region specifically.

### Genotyping

Details of genotyping, quality control, imputation methods, and genome-wide analysis for each cohort were study-specific (S1 and S2 Tables). In brief, genotyping was performed using commercially available genome-wide arrays. Imputation of single nucleotide polymorphisms (SNPs) and insertions/deletions (indels) was performed using reference panels from 1000 Genomes phase 1 version 3 or phase 3,[71] or the Haplotype Reference Consortium.[72] Analyses of UKB participants was restricted to the White British ancestry subset who self-report as White, British, and have very similar genetic ancestry backgrounds based on the results of principal components analysis (PCA); further quality control followed recommended practices for UKB[73] (S1 Table).

### Statistical analysis

We conducted genome-wide association analyses in each of the 16 cohorts, and subsequent meta-analysis of autosomal SNPs to combine results from all cohorts. Each site conducted GWAS using logistic regression models with additive genetic effects to test for associations between each variant and CBP as a binary trait. These models adjusted for age, sex, study-specific covariates, and population substructure using principal components (S2 Table). Height and body mass index (BMI), calculated as weight in kilograms divided by height in meters squared, were not included as covariates in site-specific GWAS, since these traits might lie along the causal pathway or (in the case of BMI) reflect a consequence of CBP. Harmonization and quality control of GWAS results from each cohort were conducted using the EasyQC software package in the R statistical environment (v3.2.2), using methods described previously.[74] After removal of SNPs with low minor allele frequencies (<0.005 for UKB, <0.03 for Vis, <0.01 for other cohorts) or imputation quality (<0.7 for UKB, <0.6 for other cohorts), deviation from Hardy-Weinberg equilibrium (p < 1 x 10–6), low number of cases (<15) or controls (<15), large absolute values of beta coefficient (≥10), and low minor allele count (≤10), call rate <0.95, the range of SNPs included in the meta-analysis was between 6,205,227 (Croatia-Vis) and 9,775,703 (MrOs-Gothenburg) (S2 Table). Fixed-effect inverse-variance weighted meta-analysis was performed with METAL version 2011-03-25 (http://csg.sph.umich.edu/abecasis/metal/), using the LDsr intercept as a correction factor. The meta-analysis was filtered for variants with fewer than 125,000 informative participants, to ensure that SNP-CBP associations were informed by a plurality of cohorts, and not only the UKB interim data release. For this reason, only variants with MAF<0.01 (SNPs) were included in the meta-analysis. Quality control and meta-analysis were conducted twice, independently of each other, by researchers at the University of Washington (MP and PS) and at PolyOmica (YT, YA, and LCK). The results from the two centers were compared to ensure accuracy. Q-Q and Manhattan plots were generated in R. We conducted conditional and joint (COJO) analysis using summary data (http://cnsgenomics.com/software/gcta/#About) to examine associations conditional on the most significant variant at each locus (S1 Text).

The most highly-associated variants at genome-wide significant loci were subjected to replication among UKB participants not included in the discovery sample (UKB2). Analysis in UKB2 used logistic regression with additive genetic effects, adjusting for age, sex, array, and principal components (S2 Table); significant replication was defined using a Bonferroni-corrected threshold of p<0.05 divided by the number of genome-wide significant loci. The most highly-associated variants at loci with suggestive significance were selected for a joint (discovery-replication) meta-analysis using p<5×10^−8^ to define genome-wide significance. Further details regarding analysis are provided in S1 Text. A *post hoc* analysis was conducted to stratify the discovery phase meta-analysis by the CHARGE cohorts (meta-analysis of 15 GWAS) vs. the UKB interim data release cohort (S4 Table).

For genome-wide significant variants, we examined GWAS associations with selected traits with possible links to CBP (anthropometrics, arthritis, depression and depressive symptoms, and imaging-based spinal degeneration) in publicly and privately available GWAS datasets. We conducted functional annotation using FUMA (http://fuma.ctglab.nl). FUMA draws upon multiple publicly available databases, annotating variants for consequences on gene functions using the combined annotation dependent depletion (CADD) score,[21] potential regulatory functions (RegulomeDB score),[22] and effects on gene expression using expression quantitative trait loci (eQTLs) of different tissue types (GTExv6) [23, 24] (S1 Text). The higher the CADD score, the more potentially deleterious is the variant. A CADD score of ≥10 indicates a variant predicted to be among the 10% most deleterious substitutions involving the human genome, a score of ≥20 indicates a variant among the 1% most deleterious, and so forth.[21] We used data from the Roadmap Epigenomics Project to evaluate whether the lead variants at each locus and those in LD (r^2^>0.6) reside in enhancer regions for selected tissues with possible conceptual connections to the spine via roles in chondrogenesis, vertebral development, muscle, and pain processing in the CNS.[25, 26].

Because two CBP-associated variants were found to be associated with height in prior published GWAS, we conducted *post hoc* region-specific secondary GWAS analyses accounting for height, among UKB participants from the discovery stage. Further details of functional annotation and secondary analyses are provided in S1 Text. We also conducted a two-sample Mendelian randomization to examine potential causal effects of height on CBP using significant variants associated with standing height from the GIANT consortium as the exposure, and the discovery phase meta-analysis of CBP, using the R package MRbase. We used the inverse-variance weighted regression (IVW) approach as our primary analysis method,[75] and additional analyses with other MR methods (MR-Egger regression, weighted median function, and weighted mode); presenting the results yielded from different MR methods is recommended to demonstrate sensitivity to different patterns of assumption violations.[63, 76] We examined heterogeneity among SNPs using forest plots, funnel plots, heterogeneity statistics, and the MR-Egger intercept test for directional horizontal pleiotropy. Further details of MR methods are provided in the S1 Text.

Given that 30% of UKB participants are related to at least one other person in the cohort, we also conducted post hoc secondary GWAS in UKB1 to examine whether relatedness might have influenced our results. These analyses used linear mixed-effect models (BOLT-LMM), adjusting for age, sex, study-specific covariates, and principal components. The statistical significance of GWAS results for UKB1 using BOLT-LMM were descriptively compared with the original results using logistic regression, for the lead variants achieving suggestive significance in the GWAS meta-analysis.

Finally, we used LDsr of summary-level GWAS results from the discovery stage to estimate heritability due to common autosomal SNPs and genetic correlations.[18] We transformed the observed SNP heritability to the liability scale, in order to make heritability estimates for CBP comparable with traditional heritability estimates from twin studies.[64] We used stratified LDsr to partition heritability across functional categories of the genome, using methods described previously.[65] The threshold for determining the statistical significance of 53 functional categories in partitioning heritability was set at p<9.4 x 10–4 (0.05/53). Further details of methods for partitioning heritability are provided in the S4 Appendix. We used cross-trait LDsr and publicly available meta-GWAS results from LDhub to examine genetic correlations with selected traits with possible links to CBP: anthropometrics (height, waist/hip circumference, BMI, and overweight/obesity), depression and depressive symptoms, osteoarthritis, and rheumatoid arthritis.[77, 78]

## Supporting information

S1 Table Genotyping, quality control, and imputation for each cohort. (DOCX)

S2 Table Details of the GWAS analysis for each cohort and quality control, prior to meta-analysis. (DOCX)

S3 Table Association results for meta-analysis of chronic back pain GWAS (all variants with p<5 x 10^−7^). (DOCX)

S4 Table Meta-analysis (discovery phase) results stratified by CHARGE/PainOmics cohorts vs. UKB1, and jointly. (DOCX)

S5 Table Associations between CBP-associated loci and selected phenotypes with conceptual links to CBP (anthropometrics, arthritis, depression, spinal degeneration) from prior GWAS. (DOCX)

S6 Table Variants in *CCDC26*/*GSDMC* associated with chronic back pain at the suggestive significance level (p<5 x 10^−7^) in the discovery stage meta-analysis, and associations with lumbar discectomy for sciatica in a prior GWAS. (DOCX)

S7 Table Region-specific secondary analyses accounting for height, conducted in the UKB interim data release sample. (DOCX)

S8 Table Lead variants at loci associated with chronic back pain: Comparison of results using logistic regression (PLINK) and linear mixed-effects models (BOLT- LMM). (DOCX)

S9 Table Genetic correlations between CBP and selected phenotypes of conceptual relevance to CBP, using cross-trait LD score regression. (DOCX)

S10 Table Chronic back pain definitions and related question items. (DOCX)

S1 Fig Quantile-quantile plot of p-values from the meta-analysis (discovery) of GWAS of chronic back pain (n = 158,025). *LD score regression intercept = 1.0067. GWAS = genome-wide association study.
(PDF)

S2 Fig Forest plot of rs12310519 (*SOX5*, chr12) association with chronic back pain in the meta-analysis (discovery). rs115392701 has merged into rs12310519. Point sizes are proportional to inverse variance weights. OR = odds ratio, CI = 95% confidence interval, CHS = Cardiovascular Health Study, FHS = Framingham Heart Study, GenScot = Generation Scotland, JoCo = Johnston County Osteoarthritis Project, MrOs-GBG = Mr. Os Sweden (Gothenburg), MrOs-Malmo = Mr. Os Sweden (Malmo), MrOs-US = Mr. Os United States, OAI = Osteoarthritis Initiative, RS = Rotterdam Study, SOF = Study of Osteoporotic Fractures, UK = United Kingdom, UKB = UK biobank (interim data release).
(PDF)

S3 Fig Regional association plot of *SOX5* locus. Association p-values are plotted against genomic location. Negative log of the association p-value is represented on the left-hand y-axis, and recombination rate is displayed on the right-hand y-axis. Genomic location is shown on the x-axis, chr:pos. indicated are GRCh38/hg38. RefSeq genes are indicated in the bottom panel. Linkage disequilibrium r^2^ relative to the index single nucleotide variant rs115392701 is shown using the colors in the figure legend (rs115392701 has merged into rs12310519).
(PDF)

S4 Fig Forest plot of rs1453867 (*DIS3L2*, chr2) association with chronic back pain in the meta-analysis (discovery). Point sizes are proportional to inverse variance weights. OR = odds ratio, CI = 95% confidence interval, CHS = Cardiovascular Health Study, FHS = Framingham Heart Study, GenScot = Generation Scotland, JoCo = Johnston County Osteoarthritis Project, MrOs-GBG = Mr. Os Sweden (Gothenburg), MrOs-Malmo = Mr. Os Sweden (Malmo), MrOs-US = Mr. Os United States, OAI = Osteoarthritis Initiative, RS = Rotterdam Study, SOF = Study of Osteoporotic Fractures, UK = United Kingdom, UKB = UK biobank (interim data release).
(PDF)

S5 Fig Regional association plot of *DIS3L2* locus. Association p-values are plotted against genomic location. Negative log of the association p-value is represented on the left-hand y-axis, and recombination rate is displayed on the right-hand y-axis. Genomic location is shown on the x-axis, chr:pos. indicated are GRCh38/hg38. RefSeq genes are indicated in the bottom panel. Linkage disequilibrium r^2^ relative to the index single nucleotide variant rs1453867 is shown using the colors in the figure legend.
(PDF)

S6 Fig Forest plot of rs7833174 (*CCDC26/GSDMC*, chr8) association with chronic back pain in the meta-analysis (discovery). Point sizes are proportional to inverse variance weights. OR = odds ratio, CI = 95% confidence interval, CHS = Cardiovascular Health Study, FHS = Framingham Heart Study, GenScot = Generation Scotland, JoCo = Johnston County Osteoarthritis Project, MrOs-GBG = Mr. Os Sweden (Gothenburg), MrOs-Malmo = Mr. Os Sweden (Malmo), MrOs-US = Mr. Os United States, OAI = Osteoarthritis Initiative, RS = Rotterdam Study, SOF = Study of Osteoporotic Fractures, UK = United Kingdom, UKB = UK biobank (interim data release).
(PDF)

S7 Fig Regional association plot of *CCDC26/GSDMC* locus. Association p-values are plotted against genomic location. Negative log of the association p-value is represented on the left-hand y-axis, and recombination rate is displayed on the right-hand y-axis. Genomic location is shown on the x-axis, chr:pos. indicated are GRCh38/hg38. RefSeq genes are indicated in the bottom panel. Linkage disequilibrium r^2^ relative to the index single nucleotide variant rs7833174 is shown using the colors in the figure legend.
(PDF)

S8 Fig Forest plot of rs4384683 (*DCC*, chr18) association with chronic back pain in the meta-analysis (discovery). Point sizes are proportional to inverse variance weights. OR = odds ratio, CI = 95% confidence interval, CHS = Cardiovascular Health Study, FHS = Framingham Heart Study, GenScot = Generation Scotland, JoCo = Johnston County Osteoarthritis Project, MrOs-GBG = Mr. Os Sweden (Gothenburg), MrOs-Malmo = Mr. Os Sweden (Malmo), MrOs-US = Mr. Os United States, OAI = Osteoarthritis Initiative, RS = Rotterdam Study, SOF = Study of Osteoporotic Fractures, UK = United Kingdom, UKB = UK biobank (interim data release).
(PDF)

S9 Fig Regional association plot of *DCC* locus. Association p-values are plotted against genomic location. Negative log of the association p-value is represented on the left-hand y-axis, and recombination rate is displayed on the right-hand y-axis. Genomic location is shown on the x-axis, chr:pos. indicated are GRCh38/hg38. RefSeq genes are indicated in the bottom panel. Linkage disequilibrium r^2^ relative to the index single nucleotide variant rs4384683 is shown using the colors in the figure legend.
(PDF)

S1 Text Supplemental methods. (DOCX)

S2 Text Medical ethics committee approvals. (DOCX)

S1 Appendix FUMA analyses (functional annotation). (XLSX)

S2 Appendix Instruments used in the two-sample Mendelian randomization. (XLSX)

S3 Appendix Two-sample Mendelian randomization analysis using variants associated with standing height from GIANT as the exposure, and the discovery phase meta-analysis of CBP. (DOCX)

S4 Appendix Partitioning heritability by functional category. (XLSX)
