# Supplementary material for: Genome-wide meta-analysis of 158,000 individuals of European ancestry identifies three loci associated with chronic back pain
Source: PLoS Genet. 2018 Sep 27;14(9):e1007601. doi: 10.1371/journal.pgen.1007601 (PMC6159857; doi:10.1371/journal.pgen.1007601)
Supplement: S1 Table — (DOCX) [file pgen.1007601.s001.docx]

| **Supplemental Table S1. Genotyping, quality control, and imputation for each cohort** | | | | | | | | | | | | | |
| --- | --- | --- | --- | --- | --- | --- | --- | --- | --- | --- | --- | --- | --- |
|  | **Genotyping** | | **Quality Control** | | | | | | | **Imputation** | | | |
| **Cohort** | **Platform** | **Genotype calling algorithm/ software** | **Total # of SNPs** | **SNP call rate** | **MAF** | **HWE P-value** | **ID call rate** | **Other sample QC/other exclusions** | **#SNPs that met QC** | **Imputation Software** | **Measure of Imputation Quality** | **Reference panel** | **Total # of SNPs** |
| **Discovery (Meta-analysis)** | | | | | | | | | | | | | |
| Cardiovascular Health Study (CHS) | Illumina 370CNV chip, ITMAT-Broad-CARe Illumina iSELECT chip | Illumina BeadStudio | 393,254 | ≥97% | >0% | ≥10^-5^ | > 95% | Participants excluded for presence at baseline of CHD, CHF, peripheral vascular disease, valvular heart disease, stroke, or TIA, or lack of available DNA; genotype discordant with known sex or prior genotyping. SNPs also excluded if >2 duplicate errors or Mendelian inconsistencies, heterozygote frequency = 0, SNP not found in Hapmap. | 359,953 | MaCH/ Minimac | r2 | 1000G Phase1 v3 (Nov. 2012) | 38,050,714 |
| Framingham Heart Study (FHS) | Affymetrix 500K DualGeneChip+ 50K gene-centered MIP set | BRLMM | 549,781 | ≥97% | ≥ 1% | ≥10^-6^ | ≥ 97% | Excessive heterozygosity, ethnic outliers | 412,053 | MACH/ Minimac | (O/E) σ2 ratio | 1000G Phase1 v3 (Mar. 2012) | 36,278,365 |
| Generation Scotland (GS) | Illumina OMNI_Express+Exome | Illumina GenomeStudio | 960,919 | ≥98% | ≥ 1% | ≥10^-6^ | ≥ 97% | Related individuals | 630,207 | ShapeIt2, IMPUTE2 | IMPUTE2 info score | 1000G Phase1 v3 (Mar. 2012) | 39,321,347 |
| Johnston County Osteoarthritis Project (JoCo) | Illumina Infinium 1M-Duo build 36 | Illumina Infinium 1M-Duo build 36 | 1,199,187 | ≥98% | ≥0.5% | >10^-4^ | ≥ 98% | Monomorphic SNPs, SNPs with at least one discordant genotype among duplicate samples were dropped. All subjects that were not reconcilable with the database (by sex and race) were dropped from the analysis | 934,056 | IMPUTE2 | IMPUTE2 info score | 1000G Phase 3 (Oct. 2014) | 27,547,483 |
| MrOS-Gothenburg | Illumina HumanOmni1_Quad_v1-0 B array | Illumina BeadStudio | 1,134,514 | ≥97% | > 1% | >10^-4^ | ≥ 97% | excessive autosomal heterozygozity, first and second degree relatives, genotypic sex mismatch using X and Y chromosome probe intensities and gross chromosome abnormalities | 714,543 | IMPUTE2 | IMPUTE2 info score | 1000G Phase 3 and UK10K | 35,685,596 |
| MrO- Malmo | HumanOmniExpress-12v1_B build 36 | Illumina GenomeStudio | 733,202 | ≥95% | > 1% | >10^-6^ | ≥ 97.5% | excessive autosomal heterozygozity, familial relationship (one sample excluded), genotypic sex mismatch, non-caucasians and gross chromosome abnormalities | 725,409 | IMPUTE2 | IMPUTE2 info score | 1000G Phase 3 and UK10K | 35,653,617 |
| MrOS-US | Illumina HumanOmni1_Quad_v1-0 H | Illumina GenomeStudio | 1,140,419 | ≥97% | ≥ 1% | >10^-4^ | ≥ 97% | 1) Gender mismatches; 2) ethnic outliers; 3) related individuals | 740,713 | ShapeIt2/ Minimac | MACH r2 | 1000G Phase 1 v3 | 27,438,360 |
| Osteoarthritis Initiative (OAI) | Illumina Omni-Quad 2.5M | Illumina BeadStudio | 2,440,283 | >95% | > 1% | ≥10^-6^ | ≥ 95% | Mismatches between self-reported and genetically determined gender, large chromosomal abnormalities, cryptic relatedness, ethnic outliers | 2,426,302 | MACH/ Minimac | (O/E) σ2 ratio | 1000G Phase 3 | 37,426,755 |
| Rotterdam Study 1 (RS1) | Illumina HumanHap 550K V.3 Duo | Illumina BeadStudio | 550,000 | ≥98% | ≥ 1% | ≥10^-6^ | ≥ 97.5% | 1) sex mismatch with typed X-linked markers, 2) excess autosomal heterozygosity > 0.336~ FDR > 0.1%, 3) duplicates and/or first or second degree relatives using IBS probabilities > 97% from PLINK, 4) ethnic outliers using IBS distances > 3SD from PLINK | 512,349 | MACH/ Minimac | (O/E) σ2 ratio | 1000G Phase 1 v3 | 30,072,738 |
| Rotterdam Study 2 (RS2) | Illumina HumanHap 550K V.3 Duo | Illumina BeadStudio | 550,000 | ≥98% | ≥ 1% | ≥10^-6^ | ≥ 97.5% | 1) sex mismatch with typed X-linked markers, 2) excess autosomal heterozygosity > 0.336~ FDR > 0.1%, 3) duplicates and/or first or second degree relatives using IBS probabilities > 97% from PLINK, 4) ethnic outliers using IBS distances > 3SD from PLINK | 466,389 | MACH/ Minimac | (O/E) σ2 ratio | 1000G Phase 1 v3 | 30,072,738 |
| Rotterdam Study 3 (RS3) | Illumina HumanHap 610-Quad V1 | Illumina BeadStudio | 610,901 | ≥98% | ≥ 1% | ≥10^-6^ | ≥ 97.5% | 1) sex mismatch with typed X-linked markers, 2) excess autosomal heterozygosity > 0.336~ FDR > 0.1%, 3) duplicates and/or first or second degree relatives using IBS probabilities > 97% from PLINK, 4) ethnic outliers using IBS distances > 3SD from PLINK | 517,658 | MACH/ Minimac | (O/E) σ2 ratio | 1000G Phase 1 v3 | 30,072,738 |
| Study of Osteoporotic Fractures (SOF) | Illumina HumanOmni1_Quad_v1-0 H | Illumina GenomeStudio | 1,140,420 | ≥97% | ≥ 1% | >10^-5^ | ≥ 97% | 1) Gender mismatches; 2) ethnic outliers; 3) related individuals | 740,714 | ShapeIt2/ minimac | MACH r2 | 1000G Phase 1 v3 | 27,438,360 |
| 10001 Dalmatians-Vis (Vis) | Illumina HumanHap300v1 | Illumina GenomeStudio | 317,509 | ≥98% | ≥ 1% | ≥10^-6^ | > 95% |  | 289,827 | pbwt software on the Sanger imputation server | INFO | HRC | 11,869,819 |
| 10001 Dalmatians-Korcula (Korcula) | Illumina Human370CNV-Quad | Illumina GenomeStudio | 346,034 | ≥98% | ≥ 1% | ≥10^-6^ | ≥ 97% |  | 317,898 | pbwt software on the Sanger imputation server | INFO | HRC | 11,915,415 |
| UK Biobank (UKB1) | Affymetrix UK BiLEVE Axiom array + Affymetrix UK Biobank Axiom array^*^ | Affymetrix Power Tools | 820,967 | ≥ 95% | ≥ 0.001% | ≥10^-12^ | ≥ 95% | Further QC applied by UKB is described elsewhere*. Other exclusions applied prior to analysis were inclusion of White British subset of UKB participants only (self-report of White British ancestry, with further exclusions based on PCA), exclusions for sex chromosome aneuploidy, excess of heterozygosity, and excess of relatives (having >10 third-degree relatives or closer). | 805,426 | SHAPEIT3 | MACH r2 | 1000G Phase 1 v3 + HRC | 72,355,667 |
| TwinsUK | 4 different Illumina arrays (HumanHap3001,2, HumanHap610Q, 1M‐Duo and 1.2MDuo 1M) | Illumina BeadStudio | 317,000; 616,794; 1,192,666; ~1,2 mln. respective-ly | >95% | > 1% | ≥10^-6^ | ≥ 98% | Mismatch between MZ twins and self-reported sex; non-European ancestry; related individuals | 874,733 depending on the dataset (303,940, 553,487, HumanHap1M and 1.M: 874,733) | IMPUTE2 | IMPUTE2 info score | 1000G Phase 3 | 27,445,314 |
| **Replication** | | | | | | | | | | | | | |
| UK Biobank (UKB2) | Affymetrix UK BiLEVE Axiom array + Affymetrix UK Biobank Axiom array* | Affymetrix Power Tools | 820,967 | ≥ 95% | ≥ 0.001% | ≥10^-12^ | ≥ 95% | Further QC applied by UKB is described elsewhere.* Other exclusions applied prior to analysis were inclusion of White British subset of UKB participants only (self-report of White British ancestry, with further exclusions based on PCA), exclusions for sex chromosome aneuploidy, excess of heterozygosity, and excess of relatives (having >10 third-degree relatives or closer). | 805,426 | SHAPEIT3 | MACH r2 | 1000G Phase 1 v3 + HRC | 92,693,895 |

*https://www.biorxiv.org/content/early/2017/07/20/166298.article-info and http://www.ukbiobank.ac.uk/wp-content/uploads/2014/04/UKBiobank_genotyping_QC_documentation-web.pdf
