# Supplementary material for: Genome-wide meta-analysis of 158,000 individuals of European ancestry identifies three loci associated with chronic back pain
Source: PLoS Genet. 2018 Sep 27;14(9):e1007601. doi: 10.1371/journal.pgen.1007601 (PMC6159857; doi:10.1371/journal.pgen.1007601)
Supplement: S2 Table — (DOCX) [file pgen.1007601.s002.docx]

| **Supplemental Table S2. Details of the quality control and GWAS analysis for each cohort, prior to meta-analysis^a^** | | | | | | | | | |
| --- | --- | --- | --- | --- | --- | --- | --- | --- | --- |
| **Cohort** | **Total # of SNPs post-imputation** | **Filters** | | **Model used** | **# SNPs in analysis** | **Method used to correct for genetic substructure** | **Software** | **Following file-level and meta-level quality control (described in S1 Text Supplemental Methods)^a^** | |
|  |  | **MAF** | **Imputation quality** |  |  |  |  | **# SNPs in meta-analysis** | **Genomic Inflation Factor (**λ_GC_**)** |
| **Discovery (Meta-analysis^b^)** | | | | | | | |  |  |
| Cardiovascular Health Study (CHS) | 38,050,714 | > 0% | variance on allele dosage ≤0.01 | SNP+ age + sex + clinic | 9,854,835 | NA | R | 7,528,846 | 1.00 |
| Framingham Heart Study (FHS) | 36,278,365 | > 0% | ≥0.3 | SNP + age + sex + PC1 + PC2 + PC3 + PC4 + PC5 + kinship | 19,344,131 | EIGENSTRAT | R/GEE | 7,114,199 | 1.03 |
| Generation Scotland (GS) | 39,321,347 | > 0% | none | SNP + age + sex (Unrelated individuals only) | 39,321,347 | Unrelated only | R/ProbABEL | 8,934,663 | 1.01 |
| Johnston County Osteoarthritis Project (JoCo) | 27,547,483 | > 0% | none | SNP + age + sex + PC1 + PC2 + PC3 | 27,547,483 | EIGENSOFT | PLINK | 9,236,276 | 1.02 |
| MrOS-Gothenburg | 35,685,596 | > 0% | >0.3 | SNP + age | 18,648,740 | NA | PLINK | 9,775,703 | 1.00 |
| MrO- Malmo | 35,653,617 | > 0% | >0.3 | SNP + age | 19,943,738 | NA | PLINK | 9,764,582 | 1.00 |
| MrOS-US | 27,438,360 | > 0% | none | SNP + age + clinic site + PC1 + PC2 + PC3 + PC4 | 27,438,360 | R/GWASTools package | PLINK | 8,579,487 | 1.00 |
| Osteoarthritis Initiative (OAI) | 37,426,755 | > 0% | >0.3 | SNP + age + sex + PC1 + PC2 | 8,515,357 | PLINK-MDS | PLINK | 7,552,530 | 1.01 |
| Rotterdam Study 1 (RS1) | 30,072,738 | > 0% | none | SNP+ age + sex | 30,072,738 | NA | MACH/GRIMP | 8,017,540 | 1.01 |
| Rotterdam Study 2 (RS2) | 30,072,738 | > 0% | none | SNP+ age + sex | 30,072,738 | NA | MACH/GRIMP | 7,987,628 | 1.00 |
| Rotterdam Study 3 (RS3) | 30,072,738 | > 0% | none | SNP+ age + sex | 30,072,738 | NA | MACH/GRIMP | 8,055,340 | 1.02 |
| Study of Osteoporotic Fractures (SOF) | 27,438,360 | > 0% | none | SNP + age+ clinic site + PC1 + PC2 + PC3 + PC4 | 27,438,360 | R/GWASTools package | PLINK | 8,570,668 | 1.02 |
| 10001 Dalmatians-Korcula (Korcula) | 11,915,415 | > 0% | none | SNP + age + sex + kinship (PC1 + PC2) | 11,915,415 | GenABEL kinship matrix | RegScan+SNPTEST | 7,814,464 | 1.01 |
| 10001 Dalmatians-Vis (Vis) | 11,869,819 | > 0% | none | SNP + age + sex + kinship (PC1 + PC2) | 11,869,819 | GenABEL kinship matrix | RegScan+SNPTEST | 6,205,227 | 1.01 |
| UK Biobank (UKB1)^c^ | 92,693,895 | >0.001% | none | SNP + age + sex + genotyping array type + PC1 + … + PC10 | 72,355,667^d^ | UKBB PCs | PLINK | 7,852,376 | 1.07 |
| TwinsUK | 27,445,314 | > 0% | ≥0.3 | SNP + age + sex + kinship (Unrelated individuals only) | 16,158,721 | GenABEL kinship matrix | R/GenABEL | 6,902,696 | 1.01 |
| **Replication** | | | | | | | |  |  |
| UK Biobank (UKB2)^c^ | 92,693,895 | >0.001% | none | SNP + age + sex + genotyping array type + PC1 + … + PC10 | 4^de^ | UKBB PCs | PLINK | NA | NA |

GWAS=genome-wide association study, MAF=minor allele frequency, SNP=single-nucleotide polymorphism

^a^This table presents study-level quality control prior to the GWAS of chronic back pain conducted at each site, and the GWAS analyses conducted at each site. Only participants of European ancestry were included in the site-specific GWAS. Subsequent file-level QC and meta-level QC was conducted centrally, and is described in the S1 Text Supplemental Methods. Briefly, this included filtering of SNPs with low MAF (<0.005 for UKB, <0.03 for Vis, <0.01 for other cohorts) or imputation quality (<0.7 for UKB, <0.6 for other cohorts), deviation from Hardy-Weinberg equilibrium (p < 1 x 10^-6^), low number of cases (<15) or controls (<15), large absolute values of beta coefficients (≥10), low minor allele count (≤10), and call rates <0.95.

^b^Meta-analysis LD score regression (LDSr) intercept was 1.007, indicating no population stratification. The LDSr intercept was used as a correction factor.

^c^Other exclusions applied in UKB prior to analysis were inclusion of White British subset of UKB participants only (self-report of White British, with further exclusions based on PCA), exclusions for sex chromosome aneuploidy, excess of heterozygosity, and excess of relatives (having >10 third-degree relatives or closer).

^d^only HRC-imputed SNPs were included in meta-analysis and replication, due to known problems at the time with the second set of imputed SNPs using 1000G + UK10K <http://www.ukbiobank.ac.uk/2017/07/important-note-about-imputed-genetics-data/>

^e^region-specific analyses were performed and 4 variants were included in the replication phase
