# Supplementary material for: Genome-wide meta-analysis of 158,000 individuals of European ancestry identifies three loci associated with chronic back pain
Source: PLoS Genet. 2018 Sep 27;14(9):e1007601. doi: 10.1371/journal.pgen.1007601 (PMC6159857; doi:10.1371/journal.pgen.1007601)
Supplement: S3 Table — (DOCX) [file pgen.1007601.s003.docx]

| **Supplemental Table S3:** Association Results for Meta-Analysis of Chronic Back Pain GWAS (all variants with p<5 x 10^–7^)^*^ | | | | | | | | | | | | | | |
| --- | --- | --- | --- | --- | --- | --- | --- | --- | --- | --- | --- | --- | --- | --- |
| **rsID** | **Chr:pos (hg19)** | **Effect Allele** | **Other Allele** | **EAF** | **Odds Ratio** | **r^2^ (LD)^a^** | **95% CI lower bound** | **95% CI upper bound** | **p-value** | **total N** | **MAC** | **Mean Imputation quality score** | **Het. I^2^** | **Het. p-value** |
| **rs115392701^b^** | **12:23975219** | **t** | **c** | **0.16** | **1.08** | **ref^c^** | **1.06** | **1.11** | **7.2e-10** | **155961** | **49241** | **0.94** | **0** | **0.95** |
| rs56290807 | 12:23972014 | a | c | 0.17 | 1.08 | 0.93 | 1.06 | 1.1 | 8.4e-10 | 152511 | 50963 | 0.93 | 0 | 0.78 |
| rs7134575 | 12:23978200 | a | g | 0.13 | 1.09 | 0.79 | 1.06 | 1.11 | 1.7e-09 | 154152 | 39813 | 0.94 | 0 | 0.91 |
| rs139555077 | 12:23975953 | t | g | 0.16 | 1.08 | 0.98 | 1.05 | 1.11 | 1.8e-09 | 153145 | 48325 | 0.93 | 0 | 0.92 |
| rs11831278 | 12:23960729 | t | c | 0.16 | 1.08 | 0.98 | 1.05 | 1.1 | 4.7e-09 | 153132 | 47303 | 0.93 | 0 | 0.87 |
| rs9804988 | 12:23982559 | t | c | 0.13 | 1.08 | 0.81 | 1.06 | 1.11 | 5.7e-09 | 155228 | 40367 | 0.94 | 0 | 0.92 |
| rs12581539 | 12:23977474 | t | g | 0.13 | 1.08 | 0.81 | 1.06 | 1.11 | 7.2e-09 | 153854 | 40269 | 0.93 | 0 | 0.9 |
| rs7977132 | 12:23936398 | a | g | 0.16 | 1.07 | 0.94 | 1.05 | 1.1 | 4.9e-08 | 150084 | 47947 | 0.92 | 0 | 0.85 |
| rs34616559 | 12:23947697 | t | c | 0.16 | 1.07 | 0.96 | 1.05 | 1.1 | 5.2e-08 | 152008 | 47109 | 0.92 | 0 | 0.85 |
| rs150677659 | 12:23974404 | c | g | 0.23 | 1.06 | 0.67 | 1.04 | 1.08 | 6.8e-08 | 151984 | 69856 | 0.94 | 0 | 0.65 |
| **rs1453867** | **2:232917899** | **t** | **c** | **0.65** | **0.95** | **ref^d^** | **0.93** | **0.97** | **7.7e-08** | **157283** | **111377** | **1.00** | **12.7** | **0.31** |
| **rs7833174** | **8:130718772** | **t** | **c** | **0.77** | **1.06** | **ref^e^** | **1.04** | **1.08** | **1.0e-07** | **156963** | **71950** | **0.99** | **0** | **0.7** |
| rs6651255 | 8:130723792 | t | c | 0.77 | 1.06 | 1.0 | 1.04 | 1.08 | 1.3e-07 | 157561 | 72663 | 0.99 | 0 | 0.66 |
| rs2955527 | 12:23973837 | t | g | 0.77 | 0.94 | 0.66 | 0.92 | 0.96 | 1.4e-07 | 152001 | 70879 | 0.93 | 0 | 0.49 |
| rs2955526 | 12:23973832 | a | c | 0.23 | 1.06 | 0.66 | 1.04 | 1.08 | 1.4e-07 | 152014 | 70841 | 0.93 | 0 | 0.49 |
| rs7816342 | 8:130719623 | a | g | 0.23 | 0.94 | 1.0 | 0.92 | 0.97 | 1.5e-07 | 157509 | 72350 | 0.99 | 0 | 0.67 |
| rs4130415 | 8:130718712 | t | c | 0.77 | 1.06 | 1.0 | 1.04 | 1.08 | 1.8e-07 | 157223 | 72113 | 0.99 | 0 | 0.66 |
| rs2633265 | 2:233122885 | t | c | 0.36 | 1.05 | 0.64 | 1.03 | 1.07 | 2.6e-07 | 156856 | 112305 | 0.99 | 0.7 | 0.44 |
| rs3116235 | 2:232911582 | t | c | 0.66 | 0.95 | 0.76 | 0.93 | 0.97 | 2.6e-07 | 157309 | 108326 | 1.00 | 20.4 | 0.22 |
| rs1078093 | 2:233087245 | a | g | 0.36 | 1.05 | 0.73 | 1.03 | 1.07 | 3.0e-07 | 156086 | 112722 | 0.98 | 11.2 | 0.33 |
| rs7815955 | 8:130719567 | a | t | 0.8 | 1.06 | 0.88 | 1.04 | 1.08 | 3.1e-07 | 157594 | 62913 | 0.99 | 8.2 | 0.36 |
| **rs4384683** | **18:50379032** | **a** | **g** | **0.54** | **0.95** | **ref^f^** | **0.94** | **0.97** | **3.2e-07** | **156611** | **144334** | **0.99** | **0** | **0.86** |
| rs10956487 | 8:130717716 | a | g | 0.77 | 1.06 | 1.0 | 1.04 | 1.08 | 3.4e-07 | 156089 | 71572 | 0.99 | 0 | 0.73 |
| rs4733724 | 8:130723728 | a | g | 0.8 | 1.06 | 0.88 | 1.04 | 1.08 | 3.5e-07 | 157797 | 63316 | 0.99 | 9.9 | 0.34 |
| rs6470764 | 8:130725665 | t | c | 0.2 | 0.94 | 0.87 | 0.92 | 0.97 | 3.8e-07 | 157964 | 63305 | 0.99 | 6.9 | 0.38 |
| rs923333 | 2:233082988 | t | c | 0.61 | 0.95 | 0.63 | 0.93 | 0.97 | 4.0e-07 | 155426 | 122181 | 0.97 | 2.3 | 0.43 |
| rs3116167 | 2:232988345 | a | g | 0.36 | 1.05 | 0.77 | 1.03 | 1.07 | 4.0e-07 | 157682 | 112833 | 1.00 | 16.5 | 0.27 |
| rs6725657 | 2:232812422 | a | g | 0.37 | 1.05 | 0.75 | 1.03 | 1.07 | 4.7e-07 | 155357 | 115456 | 0.96 | 17.7 | 0.25 |
| rs7826493 | 8:130738972 | a | g | 0.8 | 1.06 | 0.87 | 1.04 | 1.08 | 4.9e-07 | 157571 | 62787 | 1.00 | 14.5 | 0.29 |

*Meta-analysis results using linkage disequilibrium score regression (LDSR) intercept as a correction factor

The lead SNP in each region is indicated **in bold.** The bold line indicates the threshold for genome-wide significance (p<5 x 10^–8^)

GWAS=genome-wide association study; chr:pos= chromosome:position, EAF=effect allele frequency (weighted mean across all studies), CI=confidence interval, MAC=minor allele count, Het.=heterogeneity

^a^r^2^ with the lead SNP in the region, calculated from 1000 Genomes Phase 3 v5 GBR and CEU populations with LDlink. https://analysistools.nci.nih.gov/LDlink/

^b^rs115392701 has merged into rs12310519

^c^lead SNP for the locus on chromosome 12; the r^2^ presented in this table for other variants on chromosome 12 are calculated in relation to this SNP

^d^lead SNP for the locus on chromosome 2

^e^lead SNP for the locus on chromosome 8; the r^2^ presented in this table for other variants on chromosome 8 are calculated in relation to this SNP

^f^lead SNP for the locus on chromosome 18
