# Supplementary material for: Genome-wide meta-analysis of 158,000 individuals of European ancestry identifies three loci associated with chronic back pain
Source: PLoS Genet. 2018 Sep 27;14(9):e1007601. doi: 10.1371/journal.pgen.1007601 (PMC6159857; doi:10.1371/journal.pgen.1007601)
Supplement: S4 Table — (DOCX) [file pgen.1007601.s004.docx]

| **S4 Table.** Meta-analysis (Discovery Phase) Results Stratified by CHARGE/PainOmics Cohorts vs. UKB1, and Jointly | | | | | | | | | | | | |
| --- | --- | --- | --- | --- | --- | --- | --- | --- | --- | --- | --- | --- |
|  | | | | | **Meta-analysis of the**  **15 CHARGE/PainOmics Cohorts**  **from the Discovery Phase**  (n=38,001) | | | | **UKB1 (Interim Data Release)^1^**  (n=120,024) | | **Joint meta-analysis of CHARGE/PainOmics cohorts + UKB1**  **(the complete Discovery Phase analysis)**  (n=158,025) | |
| SNP rsID | Chr:Pos | Nearest Gene | Alleles | EAF | OR | p-value | I^2^ | Het. p-value | OR | p-value | OR | p-value |
| rs12310519^a^ | 12:23975219 | *SOX5* | T/C | 0.16 | 1.09 | 0.0013 | 0 | 0.93 | 1.08 | 1.3 x 10^-7^ | 1.08 | 7.2 x 10^-10^ |
| rs1453867 | 2:232917899 | *DIS3L2* | T/C | 0.65 | 0.96 | 0.023 | 17 | 0.26 | 0.95 | 8.7 x 10^-7^ | 0.95 | 7.7 x 10^-8^ |
| rs7833174 | 8:130718772 | *CCDC26/*  *GSDMC* | T/C | 0.77 | 1.06 | 0.022 | 0 | 0.63 | 1.06 | 2.7 x 10^-6^ | 1.06 | 1.0 x 10^-7^ |
| rs4384683 | 18:50379032 | *DCC* | A/G | 0.54 | 0.96 | 0.019 | 0 | 0.83 | 0.95 | 2.7 x 10^-6^ | 0.95 | 3.2 x 10^-7^ |

CHARGE= Cohorts for Heart and Aging Research in Genomic Epidemiology, UKB1= UK Biobank participants from the interim data release^1^, chr:pos=chromosome:position (GRCh37/hg19), Alleles=effect/other, EAF=effect allele frequency OR=odds ratio, het.=heterogeneity

^a^rs115392701 has merged into rs12310519

**1.** UK Biobank: Genotype imputation and genetic association studies of UK Biobank Interim Data Release, May 2015, 2015.
