# Supplementary material for: Genome-wide meta-analysis of 158,000 individuals of European ancestry identifies three loci associated with chronic back pain
Source: PLoS Genet. 2018 Sep 27;14(9):e1007601. doi: 10.1371/journal.pgen.1007601 (PMC6159857; doi:10.1371/journal.pgen.1007601)
Supplement: S5 Table — (DOCX) [file pgen.1007601.s005.docx]

| **Supplemental Table S5:** Associations between CBP-associated loci and selected phenotypes with conceptual links to CBP (anthropometrics, arthritis, depression, spinal degeneration) from prior GWAS* | | | | | | | | | | | | | | |
| --- | --- | --- | --- | --- | --- | --- | --- | --- | --- | --- | --- | --- | --- | --- |
| Phenotype | Cohort, consortium, or first author/ PMID (where available) | | rsID | Chr | | Pos | Eff. | Oth. | EAF | r^2**^ | Beta | SE | p-value | Total N or N cases/N controls |
| **Associations of rs12310519^a^ (*SOX5)* with phenotypes with conceptual links to CBP^b^** | | | | | | | | | | | | | | |
| **Anthropometrics** | | | | | | | | | | | | | | |
| BMI | UKB^c^ | | rs12310519 | 12 | | 23975219 | T | C | 0.14 | NA | -3.1E-04 | 0.003 | 9.2E-01 | 336,107 |
| Height | UKB^c^ | | rs12310519 | 12 | | 23975219 | T | C | 0.14 | NA | 0.007 | 0.002 | 2.2E-03 | 336,474 |
| Hip circumference | UKB^c^ | | rs12310519 | 12 | | 23975219 | T | C | 0.14 | NA | 0.007 | 0.003 | 2.2E-02 | 336,601 |
| Obesity class 1 | GIANT/ 23563607^1^ | | rs12310519 | 12 | | 23975219 | T | C | 0.14 | NA | -0.017 | 0.020 | 4.0E-01 | 29,734/61,157 |
| Obesity class 2 | GIANT/ 23563607^1^ | | rs12310519 | 12 | | 23975219 | T | C | 0.14 | NA | -5.0E-04 | 0.033 | 9.9E-01 | 7,671/49,798 |
| Obesity class 3 | GIANT/ 23563607^1^ | | rs12310519 | 12 | | 23975219 | T | C | 0.14 | NA | 0.068 | 0.067 | 3.1E-01 | 1,530/30,151 |
| Overweight | GIANT/ 23563607 | | rs12310519 | 12 | | 23975219 | T | C | 0.14 | NA | -0.017 | 0.014 | 2.2E-01 | 87,275/63,285 |
| Waist circumference | UKB^c^ | | rs12310519 | 12 | | 23975219 | T | C | 0.14 | NA | 0.002 | 0.003 | 4.9E-01 | 336,639 |
| **Arthritis** | | | | | | | | | | | | | | |
| Osteoarthritis, hip (ICD10:M16) | UKB^c^ | | rs12310519 | 12 | | 23975219 | T | C | 0.14 | NA | 3.6E-04 | 4.0E-04 | 3.6E-01 | 4,934/332,265 |
| Osteoarthritis, hip (minimal joint space width) | Castaño-Betancourt/ 24757145^2^ | | rs9804988 | 12 | | 23982559 | T | C | 0.12^d^ | 0.81 | -3.0E-04 | 0.020 | 9.9E-01 | 13,013 |
| Osteoarthritis, hip (radiographic) | TREAT-OA^3^/ 24757145 | | rs9804988 | 12 | | 23982559 | T | C | 0.12^d^ | 0.81 | 0.020 | 0.042 | 6.4E-01 | 4,349/17,836 |
| Osteoarthritis of the knee (ICD10:M17) | UKB^c^ | | rs12310519 | 12 | | 23975219 | T | C | 0.14 | NA | 1.3E-03 | 4.6E-04 | 4.2E-03 | 6,560/330,639 |
| Osteoarthritis, knee (radiographic) | TREAT-OA^3^/ 24757145 | | rs9804988 | 12 | | 23982559 | T | C | 0.12^d^ | 0.81 | 0.049 | 0.039 | 2.1E-01 | 5,636/16,972 |
| Rheumatoid arthritis | Okada/ 24390342^4^ | | rs12310519 | 12 | | 23975219 | T | C | 0.14 | NA | 0.030 | 0.031 | 3.9E-01 | 29,880/73,758 |
| Self-reported osteoarthritis | UKB^c^ | | rs12310519 | 12 | | 23975219 | T | C | 0.14 | NA | 0.002 | 9.1E-04 | 7.7E-02 | 28,257/308,902 |
| **Depression** | | | | | | | | | | | | | | |
| Depressive symptoms | SSGAC/ 27089181^5^ | | rs12310519 | 12 | | 23975219 | T | C | 0.14 | NA | 0.008 | 0.005 | 9.1E-02 | 161,460 |
| Major depressive disorder | PGC/  22472876^6^ | | rs12310519 | 12 | | 23975219 | T | C | 0.14 | NA | -0.018 | 0.011 | 1.0E-01 | 59,851/113,154 |
| Self-reported depression | UKB^c^ | | rs12310519 | 12 | | 23975219 | T | C | 0.14 | NA | 8.9E-04 | 7.7E-04 | 2.4E-01 | 19,195/317,964 |
| **Spinal degeneration** | | | | | | | | | | | | | | |
| Lumbar intervertebral disc degeneration (imaging-detected) | Williams/ 22993228^7^ | | rs12310519 | 12 | | 23975219 | T | C | 0.14 | NA | 0.114^e^ | NA | 1.1E-04 | 4,600 |
| **rs7833174 (*CCDC26/GSDMC)* with phenotypes with conceptual links to CBP^f^** | | | | | | | | | | | | | | |
| **Anthropometrics** | | | | | | | | | | | | | | |
| BMI | UKB^c^ | rs7833174 | | 8 | 130718772 | | T | C | 0.72 | NA | -0.006 | 0.003 | 4.0E-02 | 336,107 |
| Height | UKB^c^ | rs7833174 | | 8 | 130718772 | | T | C | 0.72 | NA | 0.033 | 0.002 | 1.3E-59 | 336,474 |
| Hip circumference | UKB^c^ | rs7833174 | | 8 | 130718772 | | T | C | 0.72 | NA | 0.012 | 0.003 | 1.8E-05 | 336,601 |
| Obesity class 1 | GIANT/ 23563607^1^ | rs7833174 | | 8 | 130718772 | | T | C | 0.72 | NA | -0.031 | 0.015 | 4.6E-02 | 32,280/64,407 |
| Obesity class 2 | GIANT/ 23563607^1^ | rs7833174 | | 8 | 130718772 | | T | C | 0.72 | NA | -0.001 | 0.024 | 9.7E-01 | 9,437/56,867 |
| Obesity class 3 | GIANT/ 23563607^1^ | rs7833174 | | 8 | 130718772 | | T | C | 0.72 | NA | 0.006 | 0.046 | 9.0E-01 | 1,930/33,844 |
| Overweight | GIANT/ 23563607 | rs7833174 | | 8 | 130718772 | | T | C | 0.72 | NA | -0.020 | 0.011 | 6.6E-02 | 92,059/65,656 |
| Waist circumference | UKB^c^ | rs7833174 | | 8 | 130718772 | | T | C | 0.72 | NA | 0.003 | 0.003 | 1.9E-01 | 336,639 |
| **Arthritis** | | | | | | | | | | | | | | |
| Osteoarthritis, hip (ICD10:M16) | UKB^c^ | rs7833174 | | 8 | 130718772 | | T | C | 0.72 | NA | 0.001 | 3.5E-04 | 2.8E-02 | 4,934/332,265 |
| Osteoarthritis, hip (minimal joint space width) | Castaño-Betancourt/ 24757145^2^ | rs7833174 | | 8 | 130718772 | | T | C | 0.72 | NA | 0.004 | 0.015 | 7.9E-01 | 13,013 |
| Osteoarthritis, hip (radiographic) | TREAT-OA^3^/ 24757145 | rs7833174 | | 8 | 130718772 | | T | C | 0.72 | NA | 0.104 | 0.0298 | 4.9E-04 | 4,349/17,836 |
| Osteoarthritis of the knee (ICD10:M17) | UKB^c^ | rs7833174 | | 8 | 130718772 | | T | C | 0.72 | NA | 0.000 | 3.99E-04 | 7.7E-01 | 6,560/330,639 |
| Osteoarthritis, knee (radiographic) | TREAT-OA^3^/ 24757145 | rs7833174 | | 8 | 130718772 | | T | C | 0.72 | NA | 0.040 | 0.028 | 1.5E-01 | 5,636/16,972 |
| Rheumatoid arthritis | Okada/ 24390342^4^ | rs7833174 | | 8 | 130718772 | | T | C | 0.72 | NA | 0.010 | 0.026 | 7.3E-01 | 29,880/73,758 |
| Self-reported osteoarthritis | UKB^c^ | rs7833174 | | 8 | 130718772 | | T | C | 0.72 | NA | 4.6E-04 | 8.0E-04 | 5.6E-01 | 28,257/308,902 |
| **Depression** | | | | | | | | | | | | | | |
| Depressive symptoms | SSGAC/ 27089181^5^ | rs7833174 | | 8 | 130718772 | | T | C | 0.72 | NA | 0.001 | 0.004 | 8.2E-01 | 161,460 |
| Major depressive disorder | PGC/  22472876^6^ | rs7833174 | | 8 | 130718772 | | T | C | 0.72 | NA | 0.001 | 0.011 | 9.2E-01 | 45,591/97,674 |
| Self-reported depression | UKB^c^ | rs7833174 | | 8 | 130718772 | | T | C | 0.72 | NA | 0.001 | 6.7E-04 | 7.8E-02 | 19,195/317,964 |
| **Spinal degeneration** | | | | | | | | | | | | | | |
| Lumbar intervertebral disc degeneration (imaging-detected) | Williams/ 22993228^7^ | rs7833174 | | 8 | 130718772 | | T | C | 0.72 | NA | 0.030^e^ | NA | 2.0E-01 | 4,600 |
| **rs4384683 (*DCC*) with phenotypes with conceptual links to CBP^g^** | | | | | | | | | | | | | | |
| **Anthropometrics** | | | | | | | | | | | | | | |
| BMI | UKB^c^ | rs4384683 | | 18 | 50379032 | | A | G | 0.55 | NA | -0.004 | 0.002 | 1.2E-01 | 336,107 |
| Height | UKB^c^ | rs4384683 | | 18 | 50379032 | | A | G | 0.55 | NA | -0.004 | 0.002 | 3.9E-02 | 336,474 |
| Hip circumference | UKB^c^ | rs4384683 | | 18 | 50379032 | | A | G | 0.55 | NA | -0.004 | 0.002 | 3.9E-02 | 336,601 |
| Obesity class 1 | GIANT/ 23563607^1^ | rs4384683 | | 18 | 50379032 | | A | G | 0.55 | NA | -0.014 | 0.013 | 2.6E-01 | 32,177/64,927 |
| Obesity class 2 | GIANT/ 23563607^1^ | rs4384683 | | 18 | 50379032 | | A | G | 0.55 | NA | -0.029 | 0.019 | 1.3E-01 | 9,713/61,657 |
| Obesity class 3 | GIANT/ 23563607^1^ | rs4384683 | | 18 | 50379032 | | A | G | 0.55 | NA | -0.037 | 0.034 | 2.8E-01 | 2,825/45,159 |
| Overweight | GIANT/ 23563607 | rs4384683 | | 18 | 50379032 | | A | G | 0.55 | NA | -0.011 | 0.009 | 2.3E-01 | 90,986/64,928 |
| Waist circumference | UKB^c^ | rs4384683 | | 18 | 50379032 | | A | G | 0.55 | NA | 0.002 | 0.003 | 4.9E-01 | 336,639 |
| **Arthritis** | | | | | | | | | | | | | | |
| Osteoarthritis, hip (ICD10:M16) | UKB^c^ | rs4384683 | | 18 | 50379032 | | A | G | 0.55 | NA | -3.7E-04 | 2.9E-04 | 2.0E-01 | 4,934/332,265 |
| Osteoarthritis, hip (minimal joint space width) | Castaño-Betancourt/ 24757145^2^ | rs4384683 | | 18 | 50379032 | | A | G | 0.55 | NA | 0.010 | 0.013 | 4.4E-01 | 13,013 |
| Osteoarthritis, hip (radiographic) | TREAT-OA^3^/ 24757145 | rs4384683 | | 18 | 50379032 | | A | G | 0.55 | NA | -0.003 | 0.025 | 9.0E-01 | 4,349/17,836 |
| Osteoarthritis of the knee (ICD10:M17) | UKB^c^ | rs4384683 | | 18 | 50379032 | | A | G | 0.55 | NA | -2.5E-04 | 3.4E-04 | 4.6E-01 | 6,560/330,639 |
| Osteoarthritis, knee (radiographic) | TREAT-OA^3^/ 24757145 | rs4384683 | | 18 | 50379032 | | A | G | 0.55 | NA | -0.029 | 0.024 | 2.2E-01 | 5,636/16,972 |
| Rheumatoid arthritis | Okada/ 24390342^4^ | rs4384683 | | 18 | 50379032 | | A | G | 0.55 | NA | -0.030 | 0.015 | 1.4E-01 | 29,880/73,758 |
| Self-reported osteoarthritis | UKB^c^ | rs4384683 | | 18 | 50379032 | | A | G | 0.55 | NA | -0.002 | 6.77E-04 | 1.2E-02 | 28,257/308,902 |
| **Depression** | | | | | | | | | | | | | | |
| Depressive symptoms | SSGAC/ 27089181^5^ | rs4384683 | | 18 | 50379032 | | A | G | 0.55 | NA | -0.011 | 0.003 | 5.9E-04 | 161,460 |
| Major depressive disorder | PGC/  22472876^6^ | rs4384683 | | 18 | 50379032 | | A | G | 0.55 | NA | 0.021 | 0.008 | 8.4E-03 | 59,851/113,154 |
| Self-reported depression | UKB^c^ | rs4384683 | | 18 | 50379032 | | A | G | 0.55 | NA | -0.001 | 5.7E-04 | 1.5E-01 | 19,195/317,964 |
| **Spinal degeneration** | | | | | | | | | | | | | | |
| Lumbar intervertebral disc degeneration (imaging-detected) | Williams/ 22993228^7^ | rs4384683 | | 18 | 50379032 | | A | G | 0.55 | NA | 0.006^e^ | NA | 7.7E-01 | 4,600 |

GWAS= genome-wide association study, meta-GWAS, or mega-GWAS,chr= chromosome, pos= position (hg19), EAF=effect allele frequency from Haplotype Reference Consortium, UKB= UK biobank, SSGAC= Social Science Genetic Association Consortium, GIANT= Genetic Investigation of ANthropometric Traits consortium, NA=not available, PGC=Psychiatric Genomics Consortium, TREAT-OA= Translational Research in Europe Applied Technologies for OsteoArthritis consortium

^*^Associations from publicly or privately available GWAS or meta-GWAS between the lead variants associated with back pain at genome-wide significance (p<5.0x10^-8^) in the joint meta-analysis (discovery-replication), and other phenotypes conceptually linked to CBP. If the lead variant was not present in a dataset used for look-ups, we used the variant present that was in highest LD with the lead SNP.

^**^r^2^ with the lead SNP in the region, where applicable, calculated from 1000 Genomes Phase 3 v5 GBR and CEU populations with LDlink (https://analysistools.nci.nih.gov/LDlink/). NA indicates the lead SNP in the region

^a^rs115392701 has merged into rs12310519

^b^Allele orientation presented corresponds to the direction of effect for the lead SNP rs12310519 such that *positive* beta coefficients correspond to the *same* direction of effect as the association of rs12310519 with CBP, and *negative* beta coefficients correspond to the *opposite* direction of effect as the association of rs12310519 with CBP.

^c^UKB data is available at http://www.nealelab.is/blog/2017/7/19/rapid-gwas-of-thousands-of-phenotypes-for-337000-samples-in-the-uk-biobank and https://docs.google.com/spreadsheets/d/1b3oGI2lUt57BcuHttWaZotQcI0-mBRPyZihz87Ms_No/edit?usp=sharing

^d^EAF reported in GWAS sample (where available)

^e^Direction of effect and SE not available in the dataset

^f^Allele orientation presented corresponds to the direction of effect for the lead SNP rs7833174 such that *positive* beta coefficients correspond to the *same* direction of effect as the association of rs7833174 with CBP, and *negative* beta coefficients correspond to the *opposite* direction of effect as the association of rs7833174 with CBP.

^g^Allele orientation presented corresponds to the direction of effect for the lead SNP rs4384683 such that *negative* beta coefficients correspond to the *same* direction of effect as the association of rs4384683 with CBP, and *positive* beta coefficients correspond to the *opposite* direction of effect as the association of rs4384683 with CBP. Note that this orientation is different from that for the other lead SNPs above (rs12310519 and rs7833174)

1. Berndt SI, Gustafsson S, Magi R, et al. Genome-wide meta-analysis identifies 11 new loci for anthropometric traits and provides insights into genetic architecture. *Nat Genet* 2013;45(5):501-12. doi: 10.1038/ng.2606

2. Castano-Betancourt MC, Evans DS, Ramos YF, et al. Novel Genetic Variants for Cartilage Thickness and Hip Osteoarthritis. *PLoS Genet* 2016;12(10):e1006260. doi: 10.1371/journal.pgen.1006260

3. Rodriguez-Fontenla C, Calaza M, Evangelou E, et al. Assessment of osteoarthritis candidate genes in a meta-analysis of nine genome-wide association studies. *Arthritis Rheumatol* 2014;66(4):940-9. doi: 10.1002/art.38300

4. Okada Y, Wu D, Trynka G, et al. Genetics of rheumatoid arthritis contributes to biology and drug discovery. *Nature* 2014;506(7488):376-81. doi: 10.1038/nature12873

5. Okbay A, Baselmans BM, De Neve JE, et al. Genetic variants associated with subjective well-being, depressive symptoms, and neuroticism identified through genome-wide analyses. *Nat Genet* 2016;48(6):624-33. doi: 10.1038/ng.3552

6. Major Depressive Disorder Working Group of the Psychiatric GC, Ripke S, Wray NR, et al. A mega-analysis of genome-wide association studies for major depressive disorder. *Mol Psychiatry* 2013;18(4):497-511. doi: 10.1038/mp.2012.21

7. Williams FM, Bansal AT, van Meurs JB, et al. Novel genetic variants associated with lumbar disc degeneration in northern Europeans: a meta-analysis of 4600 subjects. *Annals of the rheumatic diseases* 2013;72(7):1141-8. doi: 10.1136/annrheumdis-2012-201551
