# Supplementary material for: Genome-wide meta-analysis of 158,000 individuals of European ancestry identifies three loci associated with chronic back pain
Source: PLoS Genet. 2018 Sep 27;14(9):e1007601. doi: 10.1371/journal.pgen.1007601 (PMC6159857; doi:10.1371/journal.pgen.1007601)
Supplement: S6 Table — (DOCX) [file pgen.1007601.s006.docx]

| **Supplemental Table S6.** Variants in *CCDC26*/*GSDMC* associated with chronic back pain at the suggestive significance level (p<5 x 10^-7^) in the discovery stage meta-analysis, and associations with lumbar discectomy for sciatica in a prior GWAS* | | | | | | |
| --- | --- | --- | --- | --- | --- | --- |
|  |  | Values as reported in Bjornsdottir et al.^1^ | | | |  |
| **rsID** | **chr:pos (hg19)** | **Effect Allele** | **EAF** | **Odds ratio** | **p-value** | **Odds ratios from Bjornsdottir et al.^1^**  (reflecting allele orientation as in the current study) |
| rs6651255 | chr8:129711546 | C | 0.23 | 0.81 | 5.61E-12 | 1.23 |
| rs7833174 | chr8:129706526 | C | 0.23 | 0.81 | 5.97E-12 | 1.23 |
| rs4130415 | chr8:129706466 | C | 0.23 | 0.81 | 5.99E-12 | 1.23 |
| rs7816342 | chr8:129707377 | A | 0.23 | 0.82 | 7.81E-12 | 1.23 |
| rs7815955 | chr8:129707321 | T | 0.22 | 0.82 | 3.64E-11 | 1.23 |
| rs10956487 | chr8:129705470 | G | 0.23 | 0.82 | 1.38E-11 | 1.23 |
| rs4733724 | chr8:129711482 | G | 0.22 | 0.82 | 3.24E-11 | 1.23 |
| rs6470764 | chr8:129713419 | T | 0.22 | 0.82 | 3.79E-11 | 1.23 |
| rs7826493 | chr8:129726726 | G | 0.22 | 0.82 | 3.48E-11 | 1.23 |

Chr:pos=chromosome:position, EAF=effect allele frequency

*Bjornsdottir, 2017^1^

1. Bjornsdottir G, Benonisdottir S, Sveinbjornsson G, et al. Sequence variant at 8q24.21 associates with sciatica caused by lumbar disc herniation. *Nat Commun* 2017;8:14265. doi: 10.1038/ncomms14265
