# Supplementary material for: Genome-wide meta-analysis of 158,000 individuals of European ancestry identifies three loci associated with chronic back pain
Source: PLoS Genet. 2018 Sep 27;14(9):e1007601. doi: 10.1371/journal.pgen.1007601 (PMC6159857; doi:10.1371/journal.pgen.1007601)
Supplement: S7 Table — (DOCX) [file pgen.1007601.s007.docx]

| **Supplemental Table S7:** Region-specific secondary analyses accounting for height, conducted in the UKB interim data release sample* | | | | | | | | | | | | | | | |
| --- | --- | --- | --- | --- | --- | --- | --- | --- | --- | --- | --- | --- | --- | --- | --- |
| **Associations with CBP (for the CBP-associated lead SNPs in SOX5, *CCDC26*/*GSDMC, and DCC* from the discovery stage meta-analysis), with and without adjustment for height as a covariate** | | | | | | | | | | | | | | | |
|  |  |  |  |  |  | **Model 1** | | | | **Model 2** | | | | | |
| **rsID** | **location/gene** | **chr:pos (hg19)** | **Effect Allele** | **Other Allele** | **EAF** | **N** | **Beta** | | **p-value** | **N** | | **Beta** | | **p-value** | |
| rs12310519 | intronic/*SOX5* | 12:23975219 | T | C | 0.16 | 118108 | 0.076 | | 1.32E-07 | 117917 | | 0.076 | | 1.20E-07 | |
| rs7833174 | intergenic/ *CCDC26*/*GSDMC* | 8:130718772 | T | C | 0.77 | 119007 | 0.060 | | 2.71E-06 | 118815 | | 0.060 | | 3.37E-06 | |
| rs4384683 | intronic/*DCC* | 18:50379032 | G | A | 0.461 | 118655 | 0.050 | | 2.67E-06 | 118463 | | 0.051 | | 2.49E-06 | |
| **Associations with CBP (for the CBP-associated lead SNPs in SOX5, *CCDC26*/*GSDMC, and DCC* from region-specific analyses in UKB interim data release sample), with and without conditioning on the top height-associated variant in the region** | | | | | | | | | | | | | | | |
|  |  |  |  |  |  | **Model 1** | | | | **Model 3** | | | | | |
| **rsID** | **location/gene** | **chr:pos (hg19)** | **Effect Allele** | **Other Allele** | **EAF** | **N** | **Beta** | **p-value** | | **N** | **Beta** | | **p-value** | | **conditional rsID^b^** |
| rs7134575^a^ | intronic/*SOX5* | 12:23978200 | A | G | 0.13 | 118947 | 0.087 | 2.00E-08 | | 117309 | 0.087 | | 1.97E-08 | | rs1498873 |
| rs7833174 | intergenic/ *CCDC26*/*GSDMC* | 8:130718772 | C | T | 0.23 | 119007 | -0.060 | 2.71E-06 | | 118674 | -0.055 | | 0.11 | | rs3886937 |
| rs4384683 | intronic/*DCC* | 18:50379032 | G | A | 0.461 | 118655 | 0.050 | 2.67E-06 | | 118482 | 0.051 | | 2.62E-6 | | rs574399168 |
| **Associations with height (for the CBP-associated lead SNPs in SOX5, *CCDC26*/*GSDMC, and DCC* from region-specific analyses in UKB interim data release sample), with and without conditioning on the top CBP-associated variant in the region** | | | | | | | | | | | | | | | |
|  |  |  |  |  | **Model 4** | | | **Model 5** | | | | | | | |
| **rsID** | **location/gene** | **chr:pos (hg19)** | **Effect Allele** | **Other Allele** | **EAF** | **N** | **Beta** | **p-value** | | **N** | **Beta** | | **p-value** | | **conditional rsID^c^** |
| rs1498873 | intronic/*SOX5* | 12:24200700 | T | G | 0.22 | 118181 | 0.200 | 1.38E-10 | | 117125 | 0.19 | | 4.36E-10 | | rs7134575^a^ |
| rs3886937 | intergenic/ *CCDC26*/*GSDMC* | 8:130737391 | C | T | 0.20 | 119479 | -0.373 | 6.79E-31 | | 118482 | -0.251 | | 0.003 | | rs7833174 |
| rs574399168 | intronic/*DCC* | 18:50506453 | T | C | 0.01 | 119649 | 1.524 | 9.51eE-5 | | 118290 | 1.475 | | 1.64E-4 | | rs4384683 |
| UKB= UK biobank, CBP=chronic back pain, chr:pos=chromosome:position, EAF=effect allele frequency  *all results reflect analyses conducted in the UKB interim data release sample only  ^a^r^2^=0.90 with lead SNP rs12310519 from the discovery state meta-analysis  ^b^conditional on the top height-associated SNP in the region, listed here  ^c^conditional on the top CBP-associated SNP in the region, listed here  Model 1: CBP ~ SNP + age + sex + array + PC1 + … + PC10  Model 2: CBP ~ SNP + age + sex + + height+ array + PC1 + … + PC10  Model 3: CBP ~ SNP + age + sex + array + PC1 + … + PC10 (conditional on the top height-associated variant in the region)  Model 4: height ~ SNP + age + sex + array + PC1 + … + PC10  Model 5: height ~ SNP + age + sex + array + PC1 + … + PC10 (conditional on the top CBP-associated variant in the region | | | | | | | | | | | | | | | |
