# Supplementary material for: Genome-wide meta-analysis of 158,000 individuals of European ancestry identifies three loci associated with chronic back pain
Source: PLoS Genet. 2018 Sep 27;14(9):e1007601. doi: 10.1371/journal.pgen.1007601 (PMC6159857; doi:10.1371/journal.pgen.1007601)
Supplement: S8 Table — (DOCX) [file pgen.1007601.s008.docx]

| **Supplemental Table S8.** Lead variants at loci associated with chronic back pain: comparison of results using logistic regression (PLINK) and linear mixed-effects models (BOLT- LMM) * | | | | | | |
| --- | --- | --- | --- | --- | --- | --- |
| **rsID** | **chr:pos** | **Effect Allele** | **Other Allele** | **p-value from logistic regression analysis using PLINK** | **p-value from linear mixed-effect models using BOLT-LMM** | **Odds ratios from linear mixed-effect models using BOLT-LMM (95% CI)** |
| rs12310519 | 12:23975219 | T | C | 1.3 x 10^-7^ | 8.8 x 10^-8^ | 1.08 (1.05-1.11) |
| rs7833174 | 8:130718772 | T | C | 2.7 x 10^-6^ | 2.3 x 10^-6^ | 1.06 (1.04-1.09) |
| rs4384683 | 18:50379032 | A | G | 2.7 x 10^-6^ | 2.7 x 10^-6^ | 0.95 (0.93 - 0.97) |

Chr:pos=chromosome:position (hg19)
