# Supplementary material for: Genome-wide meta-analysis of 158,000 individuals of European ancestry identifies three loci associated with chronic back pain
Source: PLoS Genet. 2018 Sep 27;14(9):e1007601. doi: 10.1371/journal.pgen.1007601 (PMC6159857; doi:10.1371/journal.pgen.1007601)
Supplement: S9 Table — (DOCX) [file pgen.1007601.s009.docx]

| **Supplemental Table S9.** Genetic correlations between CBP and selected phenotypes of conceptual relevance to CBP, using cross-trait LD score regression* | | | | | | |
| --- | --- | --- | --- | --- | --- | --- |
| **Trait** | **PMID** | **r_g_** | **SE** | **z-score** | **p-value** | **observed h^2^** |
| **Anthropometrics** | | | | | | |
| **Waist circumference** | **25673412^1^** | **0.26** | **0.042** | **6.10** | **1.04E-09** | **0.12** |
| **Hip circumference** | **25673412^1^** | **0.21** | **0.043** | **4.91** | **8.89E-07** | **0.13** |
| **Obesity class 1** | **23563607^2^** | **0.23** | **0.047** | **4.82** | **1.44E-06** | **0.22** |
| **Waist-to-hip ratio** | **25673412^1^** | **0.19** | **0.042** | **4.49** | **7.10E-06** | **0.11** |
| **Obesity class 2** | **23563607^2^** | **0.26** | **0.059** | **4.33** | **1.46E-05** | **0.19** |
| **Obesity class 3** | **23563607^2^** | **0.31** | **0.075** | **4.09** | **4.34E-05** | **0.13** |
| **Body mass index** | **20935630^3^** | **0.17** | **0.045** | **3.90** | **9.63E-05** | **0.19** |
| **Overweight** | **23563607^2^** | **0.18** | **0.048** | **3.72** | **0.0002** | **0.11** |
| **Extreme bmi** | **23563607^2^** | **0.20** | **0.074** | **2.64** | **0.008** | **0.69** |
| Height | 20881960^4^ | 0.07 | 0.041 | 1.66 | 0.10 | 0.29 |
| Extreme waist-to-hip ratio | 23563607^2^ | 0.06 | 0.095 | 0.66 | 0.51 | 0.36 |
| **Depression-related phenotypes** | | | | | | |
| **Depressive symptoms** | **27089181^5^** | **0.52** | **0.060** | **8.66** | **4.54E-18** | **0.05** |
| **Self-reported depression** | **UK biobank** | **0.46** | **0.072** | **6.43** | **1.27E-10** | **0.02** |
| **Major depressive disorder** | **22472876^6^** | **0.48** | **0.089** | **5.37** | **7.67E-08** | **0.17** |
| **Arthritis-related phenotypes** | | | | | | |
| **Self-reported osteoarthritis** | **UK biobank** | **0.63** | **0.066** | **9.62** | **6.82E-22** | **0.02** |
| **Osteoarthritis of the knee (using ICD10 diagnostic codes)** | **UK biobank** | **0.49** | **0.085** | **5.75** | **8.67E-09** | **0.01** |
| Osteoarthritis of the hip (using ICD10 diagnostic codes) | UK biobank | 0.17 | 0.095 | 1.77 | 0.08 | 0.01 |
| Rheumatoid arthritis | 24390342^7^ | 0.11 | 0.057 | 1.96 | 0.0497 | 0.16 |

Nominally significant genetic correlations **in bold**

r_g_ reflects genetic correlations with chronic back pain (results from the discovery stage meta-analysis); p-value reflects the significance of genetic correlations with chronic back pain;observed h^2^ reflects SNP-heritability of the trait listed in each row

*CBP= chronic back pain. All GWAS results in participants of European ancestry; SNPs from the MHC (chr6 26M~34M) region was removed for all traits

1. Shungin D, Winkler TW, Croteau-Chonka DC, et al. New genetic loci link adipose and insulin biology to body fat distribution. *Nature* 2015;518(7538):187-96. doi: 10.1038/nature14132

2. Berndt SI, Gustafsson S, Magi R, et al. Genome-wide meta-analysis identifies 11 new loci for anthropometric traits and provides insights into genetic architecture. *Nat Genet* 2013;45(5):501-12. doi: 10.1038/ng.2606

3. Speliotes EK, Willer CJ, Berndt SI, et al. Association analyses of 249,796 individuals reveal 18 new loci associated with body mass index. *Nat Genet* 2010;42(11):937-48. doi: 10.1038/ng.686

4. Lango Allen H, Estrada K, Lettre G, et al. Hundreds of variants clustered in genomic loci and biological pathways affect human height. *Nature* 2010;467(7317):832-8. doi: 10.1038/nature09410

5. Okbay A, Baselmans BM, De Neve JE, et al. Genetic variants associated with subjective well-being, depressive symptoms, and neuroticism identified through genome-wide analyses. *Nat Genet* 2016;48(6):624-33. doi: 10.1038/ng.3552

6. Major Depressive Disorder Working Group of the Psychiatric GC, Ripke S, Wray NR, et al. A mega-analysis of genome-wide association studies for major depressive disorder. *Mol Psychiatry* 2013;18(4):497-511. doi: 10.1038/mp.2012.21

7. Okada Y, Wu D, Trynka G, et al. Genetics of rheumatoid arthritis contributes to biology and drug discovery. *Nature* 2014;506(7488):376-81. doi: 10.1038/nature12873
