# Supplementary material for: Genome-wide meta-analysis of 158,000 individuals of European ancestry identifies three loci associated with chronic back pain
Source: PLoS Genet. 2018 Sep 27;14(9):e1007601. doi: 10.1371/journal.pgen.1007601 (PMC6159857; doi:10.1371/journal.pgen.1007601)
Supplement: S10 Table — (DOCX) [file pgen.1007601.s010.docx]

| **Supplemental Table S10.** Chronic back pain definitions and related question items | | | |
| --- | --- | --- | --- |
| **Cohort** | **Chronic back pain definition** | **Back pain questions for each cohort** | |
| Cardiovascular Health Study | ≥1 month of back pain in consecutive years | **“*In the past year, have you had pain in your back for more than half the days of any month?*”**  “*Yes*” in ≥2 consecutive years (**cases**) vs. other combinations of non-missing responses in consecutive years (**controls**) | |
| Framingham Heart Study | ≥6 months of back pain | **“*Have you had back pain in the past 12 months?*”**  “*Most of the days*”/”*all days*” **(cases)** vs. “*no*”/”*a few days*”/”*some of the days*” **(controls)** | |
| Generation Scotland | ≥3 months of back pain | Question combinations indicating “ ***Have you been troubled by pain or discomfort*** [in the back]***, either all the time or on and off***…***for more than 3 months***?”  “*Yes*” (**cases**) vs. “*No*” (**controls**) | |
| Johnston County Osteoarthritis Project | ≥6 months of back pain | **“*On MOST days of ANY ONE MONTH in the LAST 12 MONTHS did you have pain, aching, or stiffness in any of the following?*”**  “***Lower back (L1 through S1)***” and “***middle back (thoracic)***” locations were specified.  ≥6 month duration *“with pain on most days”* **(cases)** vs. other non-missing response options **(controls)** | |
| Mr. Os Sweden | | | |
| Gothenburg | ≥6 months of back pain | **“*Have you had back pain in the past 12 months?*”**  “*Most of the time*”/”*all of the time*” **(cases)** vs. “*no*”/*”never*”/”*rarely*”/”*some of the time*” **(controls)** | |
| Malmo | ≥6 months of back pain | **“ *Have you had back pain in the past 12 months?*”**  “*Most of the time*”/”*all of the time*” **(cases)** vs. “*no*”/*”never*”/”*rarely*”/”*some of the time*” **(controls)** | |
| Mr. Os US | ≥6 months of back pain | **“*During the past 12 months have you experienced any back pain?*”**  “*Most of the time*”/”*all of the time*” **(cases)** vs. “*no*”/*”never*”/”*rarely*”/”*some of the time*” **(controls)** | |
| Osteoarthritis Initiative | ≥1 month of back pain in consecutive years | **“*How often were you bothered by back pain in the past 30 days?”***  “*All of the time*”/”*Most of the time*” in ≥2 consecutive years (**cases**) vs. any other combination of non-missing responses in consecutive years (**controls**) | |
| Rotterdam Study (RS) | | | |
| RS-1 | ≥6 months | CBP6MO |  |
| RS-1 | ≥6 months of back pain | **“*Have you had pain in the low back in the last month?”***  ≥6 month duration of low back pain **(cases)** vs. no back pain or <6 months duration low back pain **(controls)** | |
| RS-2 | ≥6 months of back pain | **“ Do you have pain or stiffness in your back?”**  ≥6 month duration **(cases)** vs. no back pain/stiffness or <6 months duration back pain/stiffness **(controls)** | |
| RS-3 | ≥6 months of back pain | **“ Do you have pain or stiffness in your back?”**  ≥6 month duration **(cases)** vs. no back pain/stiffness or <6 months duration back pain/stiffness **(controls)** | |
| Study of Osteoporotic Fractures | ≥6 months of back pain | **“*During the past 12 months have you experienced any back pain*?”**  “*Most of the time*”/”*all of the time*” **(cases)** vs. ““*no*”/*”never*”/”*rarely*”/”*some of the time*” **(controls)** | |
| 10,001 Dalmatians | | | |
| Vis | ≥3 months of back pain | **“*Have you ever had an episode of chronic LBP that lasted for over 3 months?* ”** and **“*Do you have low back pain at the moment?*”**  “*Ye*s” to both questions (**cases**) vs. “*No*” to either question (**controls**) | |
| Korcula | ≥3 months of back pain | **“*Have you ever had an episode of chronic LBP that lasted for over 3 months?* ”** and **“*Do you have low back pain at the moment?*”**  “*Ye*s” to both questions (**cases**) vs. “*No*” to either question (**controls**) | |
| TwinsUK | ≥3 months of back pain | “***In the past 3 months, have you had pain in your back on most days?”*** or  ***“Have you had pain…in this area*** [lumbar or thoracic] ***for at least the past 3 months?”***  “*Ye*s” to either question (**cases**) vs. “*No*” to both questions (**controls**) | |
| UK Biobank | ≥3 months of back pain | “***In the last month have you experienced any of the following that interfered with your usual***  ***activities?*** “ (Back pain is a response option)  **“*Have you had back pain for more than 3 months?*”**  “*Ye*s” to both questions (**cases**) vs. “*No*” to the first question or “*Ye*s” to the first question and *No*” to the second question (**controls**) | |
