# Supplementary figures and images for: Genome-wide meta-analysis of 158,000 individuals of European ancestry identifies three loci associated with chronic back pain
Source: PLoS Genet. 2018 Sep 27;14(9):e1007601. doi: 10.1371/journal.pgen.1007601 (PMC6159857; doi:10.1371/journal.pgen.1007601)

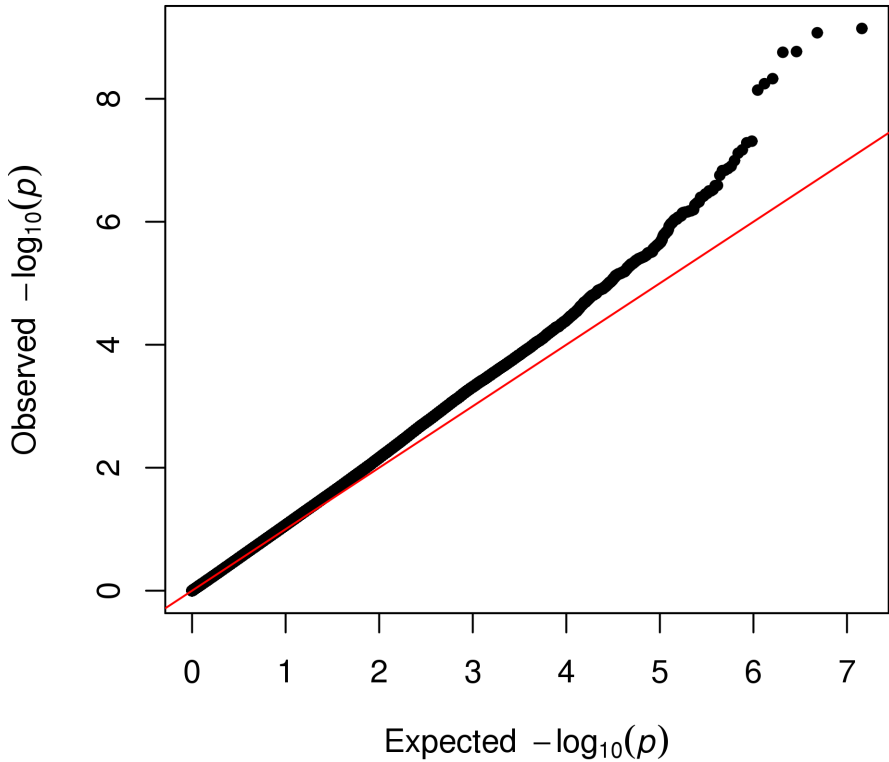

Supplement: S1 Fig — *LD score regression intercept = 1.0067. GWAS = genome-wide association study. (PDF) [file pgen.1007601.s011.pdf]

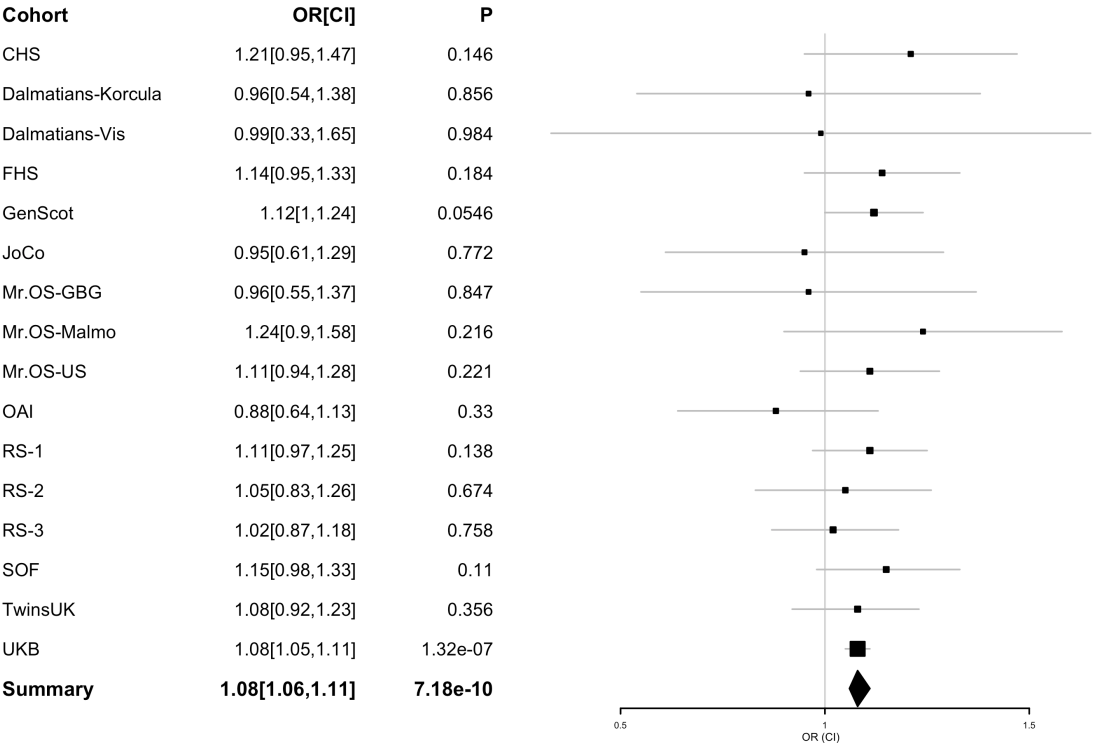

Supplement: S2 Fig — rs115392701 has merged into rs12310519. Point sizes are proportional to inverse variance weights. OR = odds ratio, CI = 95% confidence interval, CHS = Cardiovascular Health Study, FHS = Framingham Heart Study, GenScot = Generation Scotland, JoCo = Johnston County Osteoarthritis Project, MrOs-GBG = Mr. Os Sweden (Gothenburg), MrOs-Malmo = Mr. Os Sweden (Malmo), MrOs-US = Mr. Os United States, OAI = Osteoarthritis Initiative, RS = Rotterdam Study, SOF = Study of Osteoporotic Fractures, UK = United Kingdom, UKB = UK biobank (interim data release). (PDF) [file pgen.1007601.s012.pdf]

Plotted SNPs

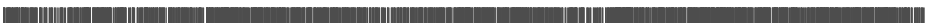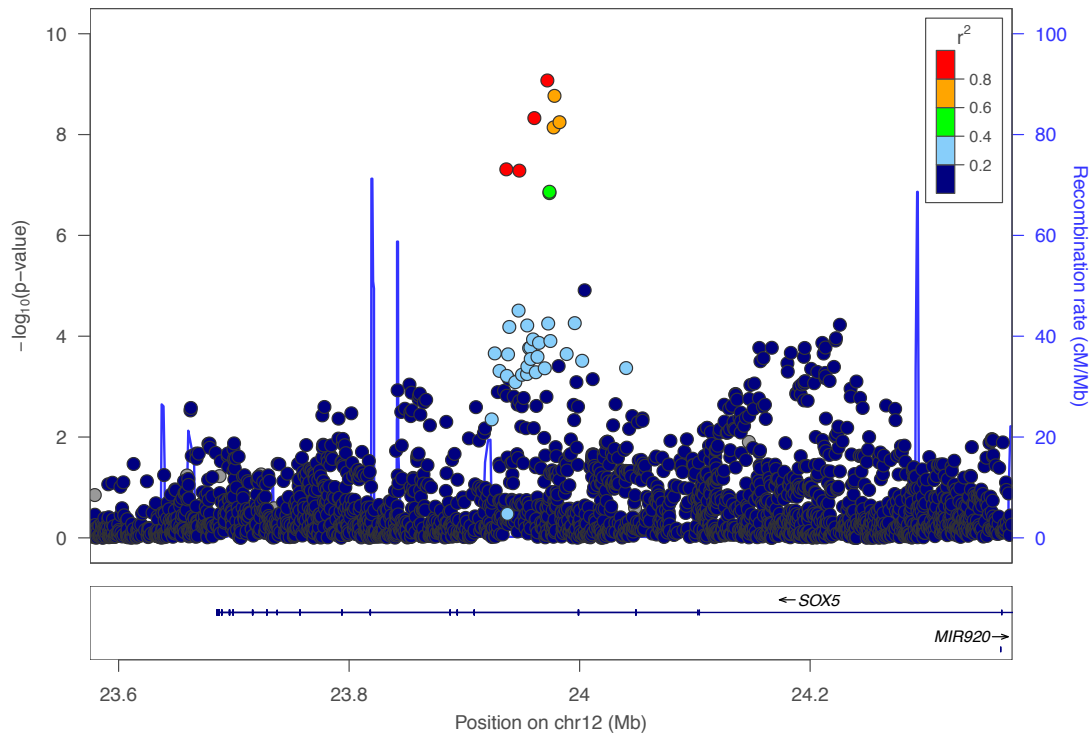

Supplement: S3 Fig — Association p-values are plotted against genomic location. Negative log of the association p-value is represented on the left-hand y-axis, and recombination rate is displayed on the right-hand y-axis. Genomic location is shown on the x-axis, chr:pos. indicated are GRCh38/hg38. RefSeq genes are indicated in the bottom panel. Linkage disequilibrium r2 relative to the index single nucleotide variant rs115392701 is shown using the colors in the figure legend (rs115392701 has merged into rs12310519). (PDF) [file pgen.1007601.s013.pdf]

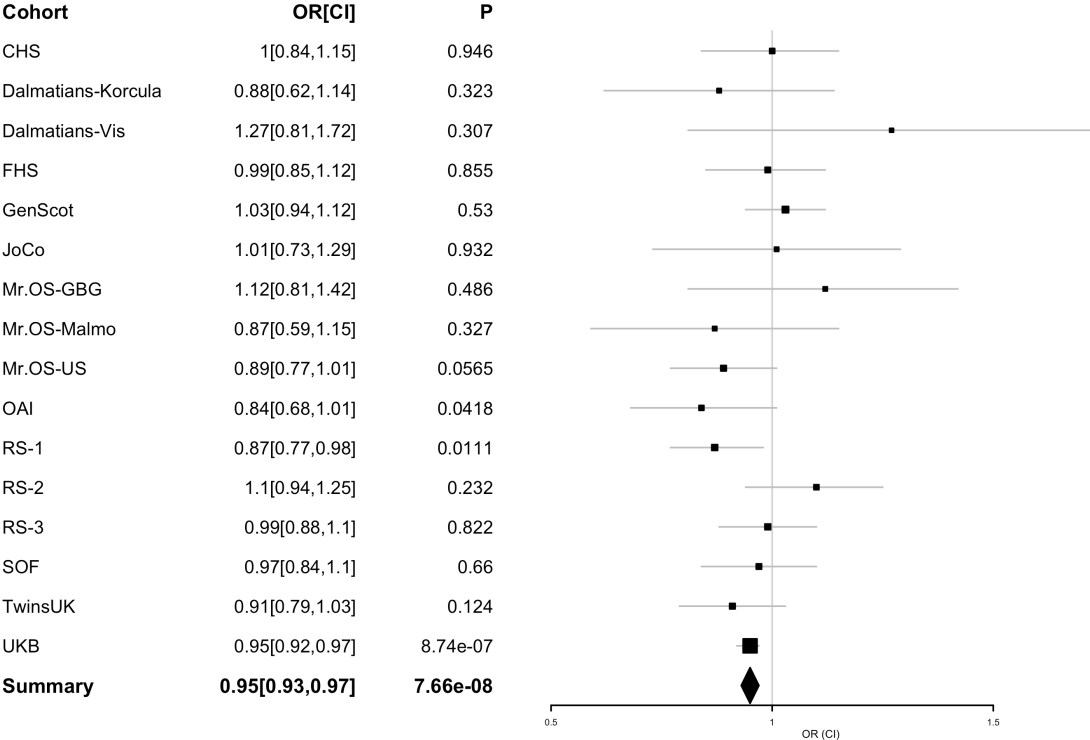

Supplement: S4 Fig — Point sizes are proportional to inverse variance weights. OR = odds ratio, CI = 95% confidence interval, CHS = Cardiovascular Health Study, FHS = Framingham Heart Study, GenScot = Generation Scotland, JoCo = Johnston County Osteoarthritis Project, MrOs-GBG = Mr. Os Sweden (Gothenburg), MrOs-Malmo = Mr. Os Sweden (Malmo), MrOs-US = Mr. Os United States, OAI = Osteoarthritis Initiative, RS = Rotterdam Study, SOF = Study of Osteoporotic Fractures, UK = United Kingdom, UKB = UK biobank (interim data release). (PDF) [file pgen.1007601.s014.pdf]

Plotted SNPs

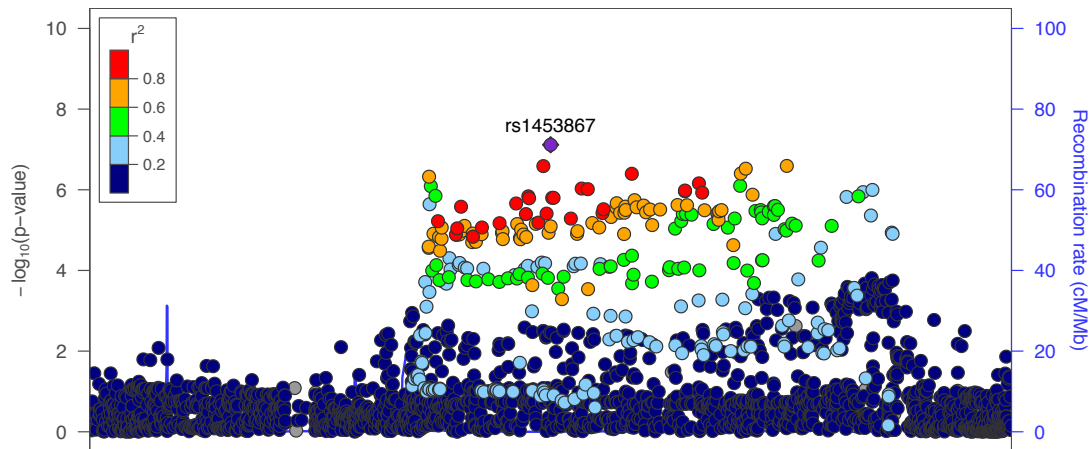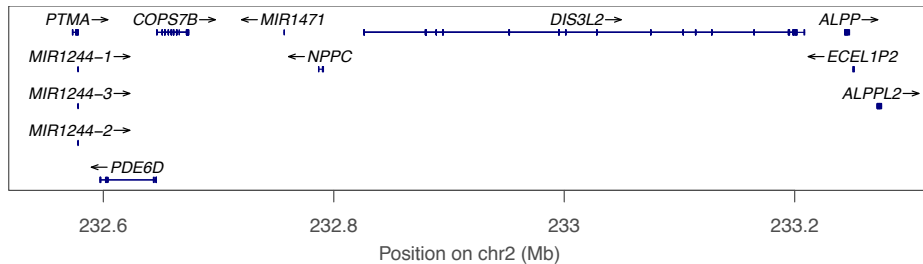

Supplement: S5 Fig — Association p-values are plotted against genomic location. Negative log of the association p-value is represented on the left-hand y-axis, and recombination rate is displayed on the right-hand y-axis. Genomic location is shown on the x-axis, chr:pos. indicated are GRCh38/hg38. RefSeq genes are indicated in the bottom panel. Linkage disequilibrium r2 relative to the index single nucleotide variant rs1453867 is shown using the colors in the figure legend. (PDF) [file pgen.1007601.s015.pdf]

Plotted SNPs

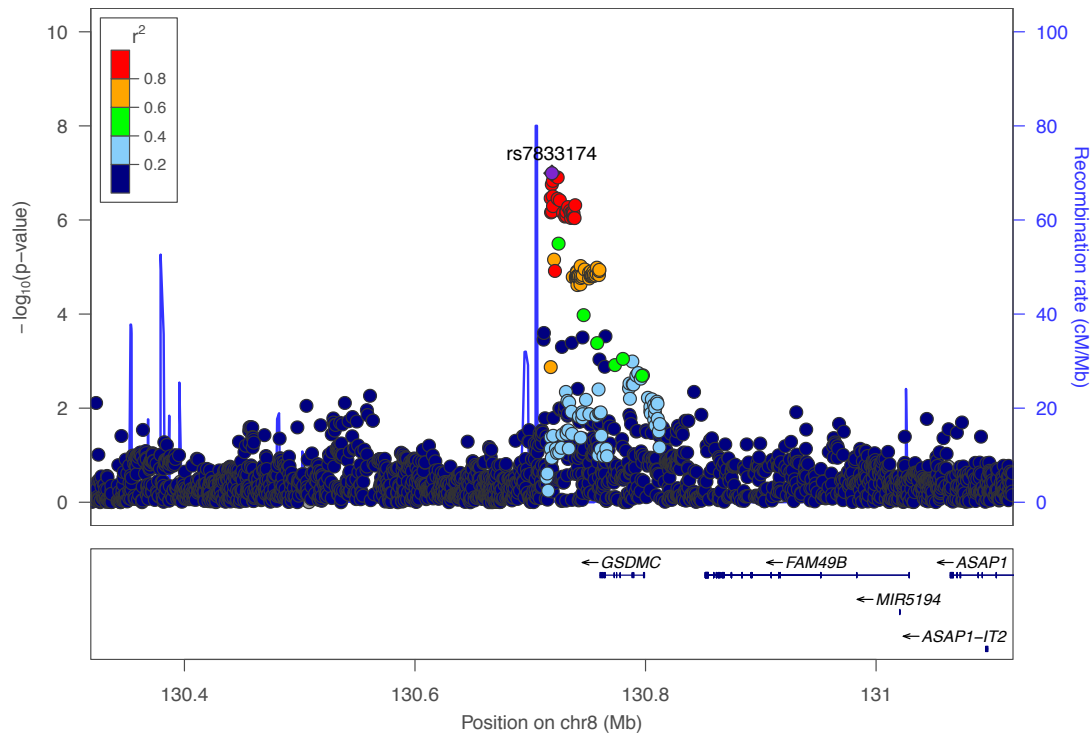

Supplement: S7 Fig — Association p-values are plotted against genomic location. Negative log of the association p-value is represented on the left-hand y-axis, and recombination rate is displayed on the right-hand y-axis. Genomic location is shown on the x-axis, chr:pos. indicated are GRCh38/hg38. RefSeq genes are indicated in the bottom panel. Linkage disequilibrium r2 relative to the index single nucleotide variant rs7833174 is shown using the colors in the figure legend. (PDF) [file pgen.1007601.s017.pdf]

Plotted SNPs

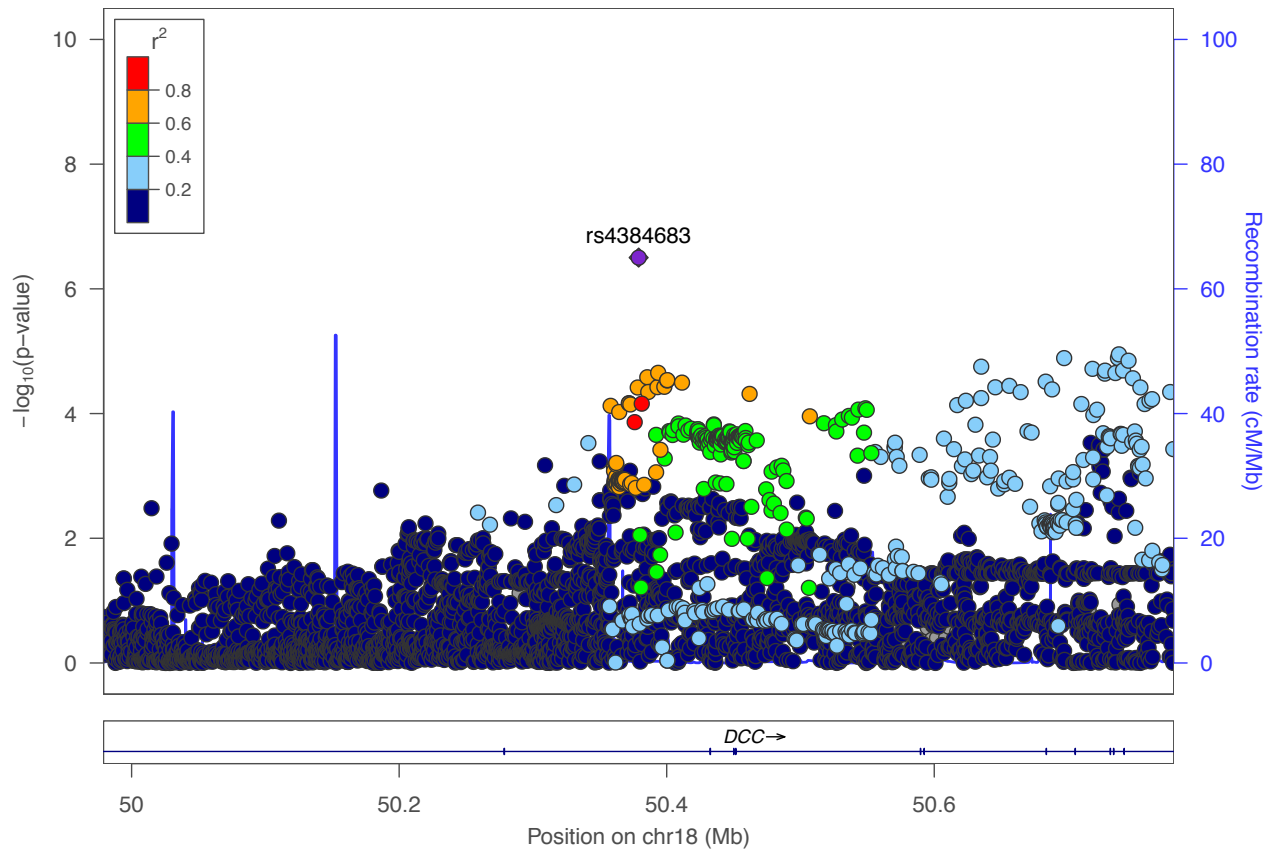

Supplement: S9 Fig — Association p-values are plotted against genomic location. Negative log of the association p-value is represented on the left-hand y-axis, and recombination rate is displayed on the right-hand y-axis. Genomic location is shown on the x-axis, chr:pos. indicated are GRCh38/hg38. RefSeq genes are indicated in the bottom panel. Linkage disequilibrium r2 relative to the index single nucleotide variant rs4384683 is shown using the colors in the figure legend. (PDF) [file pgen.1007601.s019.pdf]
