# Supplementary material for: Genome-wide meta-analysis of 158,000 individuals of European ancestry identifies three loci associated with chronic back pain
Source: PLoS Genet. 2018 Sep 27;14(9):e1007601. doi: 10.1371/journal.pgen.1007601 (PMC6159857; doi:10.1371/journal.pgen.1007601)

Cohort	OR[CI]	P
CHS	1.02[0.85,1.2]	0.796
Dalmatians-Korcula	1.35[1.06,1.64]	0.0427
Dalmatians-Vis	0.74[0.23,1.25]	0.253
FHS	1.19[1.03,1.35]	0.0288
GenScot	0.98[0.88,1.09]	0.77
JoCo	0.95[0.63,1.26]	0.746
Mr.OS-GBG	1.17[0.81,1.52]	0.393
Mr.OS-Malmo	0.91[0.58,1.23]	0.559
Mr.OS-US	1.02[0.88,1.16]	0.752
OAI	1.11[0.92,1.31]	0.291
RS-1	1.07[0.95,1.19]	0.274
RS-2	1.05[0.88,1.23]	0.562
RS-3	1.12[0.99,1.24]	0.0776
SOF	1.02[0.87,1.17]	0.79
TwinsUK	1.07[0.93,1.21]	0.343
UKB	1.06[1.04,1.09]	2.7e-06
**Summary**	**1.06[1.04,1.08]**	**1.01e-07**

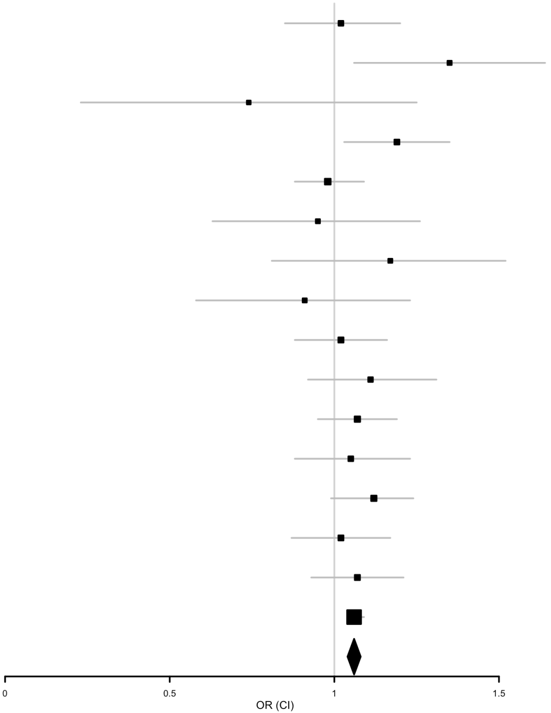

Supplement: S6 Fig — Point sizes are proportional to inverse variance weights. OR = odds ratio, CI = 95% confidence interval, CHS = Cardiovascular Health Study, FHS = Framingham Heart Study, GenScot = Generation Scotland, JoCo = Johnston County Osteoarthritis Project, MrOs-GBG = Mr. Os Sweden (Gothenburg), MrOs-Malmo = Mr. Os Sweden (Malmo), MrOs-US = Mr. Os United States, OAI = Osteoarthritis Initiative, RS = Rotterdam Study, SOF = Study of Osteoporotic Fractures, UK = United Kingdom, UKB = UK biobank (interim data release). (PDF) [file pgen.1007601.s016.pdf]
