# Supplementary material for: Genome-wide meta-analysis of 158,000 individuals of European ancestry identifies three loci associated with chronic back pain
Source: PLoS Genet. 2018 Sep 27;14(9):e1007601. doi: 10.1371/journal.pgen.1007601 (PMC6159857; doi:10.1371/journal.pgen.1007601)

Cohort	OR[CI]	P
CHS	0.95[0.8,1.11]	0.548
Dalmatians-Korcula	0.91[0.67,1.15]	0.448
Dalmatians-Vis	1.23[0.8,1.66]	0.351
FHS	1.11[0.97,1.25]	0.155
GenScot	0.95[0.86,1.04]	0.218
JoCo	1.02[0.76,1.28]	0.884
Mr.OS-GBG	0.77[0.48,1.06]	0.0808
Mr.OS-Malmo	0.89[0.62,1.15]	0.378
Mr.OS-US	0.94[0.82,1.06]	0.341
OAI	1[0.84,1.16]	0.968
RS-1	0.97[0.87,1.07]	0.618
RS-2	0.93[0.78,1.07]	0.318
RS-3	0.94[0.83,1.04]	0.212
SOF	0.95[0.83,1.08]	0.448
TwinsUK	0.98[0.86,1.09]	0.701
UKB	0.95[0.93,0.97]	2.67e-06
**Summary**	**0.95[0.94,0.97]**	**3.15e-07**

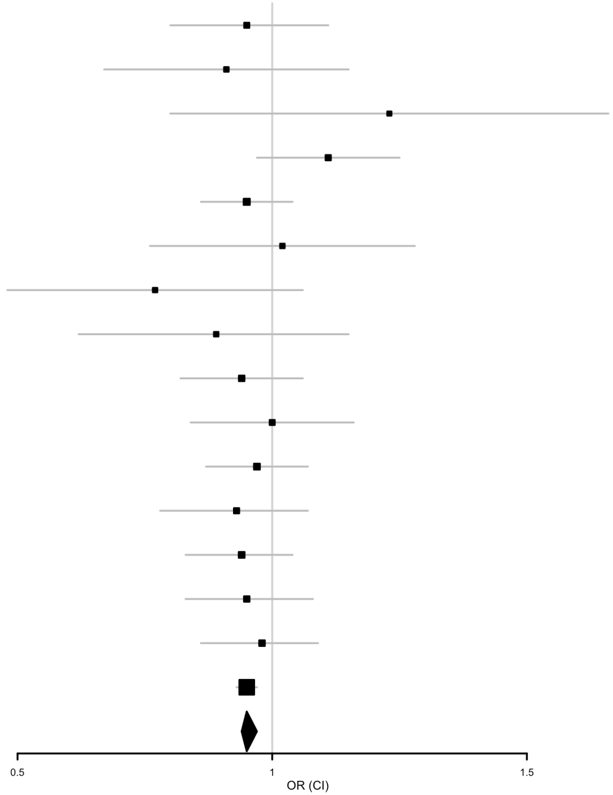

Supplement: S8 Fig — Point sizes are proportional to inverse variance weights. OR = odds ratio, CI = 95% confidence interval, CHS = Cardiovascular Health Study, FHS = Framingham Heart Study, GenScot = Generation Scotland, JoCo = Johnston County Osteoarthritis Project, MrOs-GBG = Mr. Os Sweden (Gothenburg), MrOs-Malmo = Mr. Os Sweden (Malmo), MrOs-US = Mr. Os United States, OAI = Osteoarthritis Initiative, RS = Rotterdam Study, SOF = Study of Osteoporotic Fractures, UK = United Kingdom, UKB = UK biobank (interim data release). (PDF) [file pgen.1007601.s018.pdf]
