# Supplementary material for: Genome-wide meta-analysis of 158,000 individuals of European ancestry identifies three loci associated with chronic back pain
Source: PLoS Genet. 2018 Sep 27;14(9):e1007601. doi: 10.1371/journal.pgen.1007601 (PMC6159857; doi:10.1371/journal.pgen.1007601)
Supplement: S1 Text — (DOCX) [file pgen.1007601.s020.docx]

**S1. Text: Supplemental Methods**

**Cohort Descriptions and Cohort-Specific Phenotype Descriptions**

See S8 Table for further details of chronic back pain phenotype definitions for each cohort.

**Cardiovascular Health Study (CHS)**- CHS is a community-based observational study of risk factors for cardiovascular disease in ambulatory adults 65 years or older.^1^ Participants were recruited from Medicare enrollment lists in four U.S. communities: Forsyth County, North Carolina; Sacramento County, California; Washington County, Maryland; and Pittsburgh, Pennsylvania. Starting in 1989, 5201 participants underwent extensive annual clinical examinations. An additional predominantly African-American cohort of 687 individuals was enrolled in 1992-1993. Study measures included traditional cardiovascular risk factors such as blood pressure and lipids as well as objective measures of subclinical disease, including echocardiography of the heart, carotid ultrasound, and cranial magnetic-resonance imaging. African-Americans were not included in the current study due to the meta-analysis’ focus on individuals of European ancestry only. All participants gave informed consent to use genetic data for analyses. A variety of self-report measures were also collected. Back pain questions began during the 2^nd^ examination, and included the question “In the past year, have you had pain in your back for more than half the days of any month?”.

**Framingham Heart Study (FHS)-** FHS began in 1948 as a longitudinal population-based cohort study of the causes of heart disease.^2^ The Original cohort was comprised of 5,209 men and women from the town of Framingham, Massachusetts. In 1971, 5,124 offspring of the original Framingham cohort and their spouses were entered in the Offspring cohort.^3^ Examination 8 of the Framingham Offspring cohort study began in 2005 and concluded in 2008. Participants were asked “Have you had back pain in the past 12 months?”, with response options including “no”, “a few days”, “some days”, “most of the days”, and “all days”.

**Generation Scotland (GS)-** GS is a family-based genetic epidemiology study with genetic, sociodemographic, and clinical data from approximately 24,000 volunteers across Scotland aged 18–98 years.^4^ Participants were recruited through a variety of methods from February 2006 to March 2011. Biological samples and anonymized data form a resource for research on the genetics of health, disease and quantitative traits of current and projected public health importance. A ‘broad’ form of consent was acquired from participants to use their data and samples for a wide range of medical research, including re-contact for the potential collection of other data or samples, or for participation in related studies.^4^ Participants reporting any musculoskeletal pain were asked whether specific areas of pain or discomfort had been going on for more than 3 months. Participants specified ‘yes’ or ‘no’ for back pain of duration > 3 months.

**Johnston County Osteoarthritis Project (JoCo)-** JoCo is an ongoing, population-based

prospective cohort begun in 1990 to fill knowledge gaps about prevalence, incidence, and progression of OA, and its risk factors, in Caucasian and African-American men and women in North Carolina.^5^ Participants were recruited from six townships and surrounding rural areas, and eligible individuals completed two home interviews and clinic examinations. The sample has been followed approximately every 5 years since inception with repeated surveys, radiographic, and clinical assessments, and other diagnostic testing. Beginning in 2012, participants were asked a series of questions about areas of pain experienced in the last 12 months, including middle back (thoracic) and/or lower back (L1 through S1) pain that they had experienced on most days of any one month in the last year. Those who reported pain in the middle back or lower back were then asked the number of months in the past year during which they had had pain in this location on most days of the month.

**MrOs Sweden-** MrOs Sweden is a population-based cohort study of community-living

Swedish men aged 69–81, part of a multi-national collaboration including the MrOs US study and MrOs Hong Kong.^6^ MrOs Sweden includes 3,014 men recruited from 3 study centers, including Gothenburg, Malmo, and Uppsala. Participants were randomly selected from the Swedish national population register. At the study baseline, participants answered a questionnaire including fracture history, lifestyle, and clinical symptoms.^6^ Participants were asked “Have you had back pain in the past 12 months?”. Response options included “no”, ”never”, ”rarely”, ”some of the time”, “most of the time”, and ”all of the time”. Only participants from the Gothenburg and Malmo sites who had available genetic and back pain data were included in the current study.

**MrOs US (The Osteoporotic Fractures in Men)** - MrOs US is a prospective, population-based cohort study of older men recruited from the geographic regions surrounding Birmingham, Minneapolis, Palo Alto, Pittsburgh, Portland, and San Diego. The study was designed to determine the extent to which fracture risk is related to bone mass and geometry, lifestyle, anthropometric, and neuromuscular measures, as well as to determine how fractures affect quality of life in older men.^7^ MrOs US is part of a multi-national collaboration including the MrOs Sweden study and MrOs Hong Kong. At baseline, MrOs participants completed questionnaires regarding medical history, medications, physical activity, diet, alcohol intake, and cigarette smoking. Objective measures of anthropometric, neuromuscular, vision, strength, and cognitive variables were also obtained. MrOs participants were asked the question “During the past 12 months have you experienced any back pain?”, with response options including “no”, ”never”, ”rarely”, ”some of the time”, “most of the time”, and ”all of the time”.

**Osteoarthritis Initiative (OAI)**- The OAI is a multicenter, longitudinal, prospective observational study of knee osteoarthritis. It is a public-private partnership between the NIH and private industry that seeks to develop a public domain research resource to facilitate the scientific evaluation of biomarkers for osteoarthritis as potential surrogate endpoints for disease onset and progression.^8^ OAI includes 4,796 participants age 45-79 recruited from clinical centers in Maryland, Ohio, Pennsylvania, and Rhode Island. Participants completed serial assessments and examinations annually since study inception, with a smaller subset seen more frequently for analysis of change over shorter intervals. At each annual assessment, participants were asked the question ““How often were you bothered by back pain in the past 30 days?”, with response options including “no”, “rarely”, “some of the time”, “most of the time”, and “all of the time”.

**Rotterdam Study (RS)-** Rotterdam Study is a prospective population-based cohort study on the determinants of chronic disabling diseases, ongoing since 1990.^9^ The first Rotterdam cohort (RS-1) included 7,983 persons ≥55 years of age living in the Ommoord district in the city of Rotterdam, in the Netherlands. In 2000, 3011 additional participants ≥55 years of age were added to the cohort (RS-2). In 2006, a further extension of the cohort was initiated in which 3,932 subjects living in the Ommoord district were included, aged 45–54 years (RS-3)^9^. Back pain was assessed at several of the RS examinations. In RS-1, back pain was assessed with the question, “Have you had pain in the low back in the last month?”, with further questions as to the duration of pain in the low back. In RS-2 and RS-3, back pain was assessed with the question, “Do you have pain or stiffness in your back?”, with further questions as to the duration of pain in the back.

**Study of Osteoporotic Fractures (SOF)**- SOF is a community-based multi-center prospective observational study of a cohort of 10,366 women ≥55 years of age who were recruited from four metropolitan areas in the US: Minneapolis, Pittsburgh, Portland, and Baltimore. The primary purpose of SOF was to describe the risk factors for osteoporotic fractures in women.^10^ Participants were initially recruited from mailings to age-eligible women identified from community-based listings, such as memberships of large health maintenance organizations. SOF is funded by NIA. SOF participants were asked the question “During the past 12 months have you experienced any back pain?”, with response options including “no”, ”never”, ”rarely”, ”some of the time”, “most of the time”, and ”all of the time, constantly”.

**10,001 Dalmatians-** This study aims to identify genetic and environmental determinants of

health and disease in genetic-isolate island populations from Dalmatia, Croatia. The Vis and Korcula cohorts included unselected Croatians who were recruited from villages on the Dalmatian islands of Vis and Korcula. Participants completed questionnaires regarding lifestyle and environmental exposures, biochemical and physiological measurements, and genotyping. Participants were asked the questions “Have you ever had an episode of chronic LBP that lasted for over 3 months? ” and “Do you have low back pain at the moment?”, and participants who responded yes to both questions were considered to have chronic back pain of duration > 3 months.

**TwinsUK**- TwinsUK is a British adult twin registry originally assembled to study the heritability and genetics of age-related diseases^11^. The registry was started in 1992 and consists of approximately 13,000 monozygotic and dizygotic adult Northern European twins aged 16 to 85 years from all over the UK, as well as some parents and siblings. Individuals were recruited from the general population through national media campaigns throughout the UK. TwinsUK is generally representative of singletons and the United Kingdom population. TwinsUK participants were asked the question “In the past 3 months, have you had pain in your back on most days?”. Further questions inquired about back pain in specific locations while referencing a mannekin showing locations of the pain in the thoracic and lumbar regions, and whether any such pain had persisted for at least the past 3 months.

**UK Biobank (UKB)-** The UK (United Kingdom) Biobank is a population-based prospective study involving more than 500,000 participants, established to allow detailed studies of the genetic and nongenetic determinants of the diseases of middle age and old age.^12^ UKB aims to combine extensive assessment of exposures with comprehensive follow-up and characterization of many different diseases and health-related outcomes, as well as to promote innovative science by maximizing access to the resource. The study thus far has collected and continues to collect longitudinal data and extensive phenotypic and genotypic detail about its participants, including data from questionnaires, physical measures, diagnostic imaging, and genotyping. Participants reported locations (including back pain) where they had experienced pain in the last month that interfered with usual activities. Participants reporting back pain specified ‘yes’ or ‘no’ for back pain of duration > 3 months. Participants in the current study included the White British subset of UKB participants only (self-report of White British, with further exclusions based on PCA), with further exclusions for sex chromosome aneuploidy, excess of heterozygosity, and excess of relatives (having >10 third-degree relatives or closer). For analytic purposes, White British ancestry UKB participants included in the interim data release^13^ were treated as a separate subcohort (UKB1) from those not included in the interim data release (UKB2).

**Statistical Analysis (Detailed Description)**

This subsection is a more detailed description of the Methods presented in the manuscript. Some content contained in the manscript Methods section is repeated in this subsection, so that this document functions as a stand-alone complete description of the Methods used.

Discovery meta-analysis included adults of European ancestry from the 16 population- and community-based cohorts listed above (15 cohorts from the CHARGE and PainOmics consortia, and participants from the UKB interim data release (UKB1). Replication was conducted in a sample of UKB European ancestry participants (UKB2) who were not part of the interim data release , and a joint (discovery-replication) meta-analysis was performed

For the discovery stage, we conducted genome-wide association analyses in each of the 16 cohorts, and subsequent meta-analysis of autosomal SNPs to combine results from all cohorts. Each site conducted GWAS using logistic regression models with additive genetic effects to test for associations between each variant and chronic back pain as a binary trait. These models adjusted for age, sex, and study-specific covariates such as study site, clinic site, or array (Supplemental Table S2). Each cohort evaluated population substructure and adjusted for principle components where indicated (Supplemental Table S2). Height and body mass index (BMI), calculated as weight in kilograms divided by height in meters squared, were not included as covariates in site-specific GWAS, since these traits might lie along the causal pathway or (in the case of BMI) reflect a consequence of CBP. Harmonization and quality control for GWAS results from each cohort were conducted using the EasyQC software package in the R statistical environment (v3.2.2), using methods detailed previously for file-level and meta-level quality control.^14^ After removal of SNPs with low minor allele frequencies (<0.005 for UKB, <0.03 for Vis, <0.01 for other cohorts) or imputation quality (<0.7 for UKB, <0.6 for other cohorts), deviation from Hardy-Weinberg equilibrium (p < 1 x 10-6), low number of cases (<15) or controls (<15), large absolute values of beta coefficients (≥10), and low minor allele count (≤10), call rates <0.95, the range of SNPs included in the meta-analysis was between 6,205,227 (Croatia-Vis) and 9,775,703 (MrOs-Gothenburg) (2mental Table S3). Fixed-effect inverse-variance weighted meta-analysis was performed using METAL (<http://csg.sph.umich.edu/abecasis/metal/>), using the LDsr intercept as a correction factor. The meta-analysis was filtered for variants with fewer than 125,000 informative participants, to ensure that SNP-CBP associations were informed by a plurality of cohorts, and not only the UKB interim data release. Accordingly, only variants with MAF>0.01 (SNPs) were included in the meta-analysis. Quality control and meta-analysis were conducted twice, independently of each other, by researchers at the University of Washington (MP and PS) and PainOmics (YA, LCK, and YT). The results from the two centers were compared to ensure accuracy. We used linkage disequilibrium (LD) score regression (LDsr) to examine potential confounding, using LD scores from European Ancestry 1000 Genomes data.^15^ 1000 Genomes LD scores were used given the setting of this work as a meta-analysis, where it was not feasible to take into account the LD composition of all cohorts. The genome-wide significance level was defined as p<5×10^–8^, and suggestive significance level was defined as p<5×10^–7^, after using the LDsr intercept as a correction factor. Q-Q and Manhattan plots were generated in R. Effect heterogenity across studies was quantified using the I^2^ metric (range 0-100%).^16^ Between-study heterogeneity was tested using the Cochran Q statistic which was considered significant at p<0.1. We used LocusZoom (http://csg.sph.umich.edu/locuszoom/) to evaluate regional association plots at regions of interest, and conducted Genome-Wide Complex Trait Analysis conditional and joint analyses (http://cnsgenomics.com/software/gcta/#About) for variants in each locus exceeding the suggestive significance level, conditional on the most significant variant at each locus.

The most highly associated variants at genome-wide significant or suggestive loci were subjected to replication in the remaining UKB participants not included in the discovery sample (UKB2). Analysis in UKB2 used logistic regression with additive genetic effects, adjusting for age, sex, array, and 10 principal components (Supplemental Table S2); significant replication was defined using a Bonferroni-corrected threshold of p<0.05 divided by the number of genome-wide significant loci (0.05/1). The most highly-associated variants at loci with suggestive significance were selected for a joint (discovery-replication) meta-analysis using p<5×10^–8^ to define genome-wide significance.

For genome-wide significant variants, we examined GWAS associations with selected traits with possible links to CBP (anthropometrics [BMI, height, hip circumference, obesity/overweight, waist circumference] arthritis [clinical diagnosis of hip arthritis based on ICD-10 codes, hip minimal joint space width, radiographic hip osteoarthritis, clinical diagnosis of knee arthritis based on ICD-10 codes, radiographic knee osteoarthritis], depression and depressive symptoms [depressive symptoms, major depressive disorder, self-reported depression], and imaging-based spinal degeneration [imaging-detected lumbar intervertebral disc degeneration]) in publicly and privately available GWAS datasets. We conducted functional annotation using FUMA (http://fuma.ctglab.nl). FUMA draws upon multiple publicly available databases, annotating variants for functional consequences on gene functions using the combined annotation dependent depletion (CADD) score,^17^ potential regulatory functions (RegulomeDB score),^18^ and effects on gene expression using expression quantitative trait loci (eQTLs) of different tissue types (GTExv6 and other databases for eQTLs)^19,20^ (S1 Appendix). The CADD score is computed based on 63 annotations; the higher the score, the more potentially deleterious the variant is. A CADD score of ≥10 indicates a variant predicted to be among the 10% most deleterious substitutions involving the human genome, a score of ≥20 indicates the 1% most deleterious, and soforth. We used data from the Roadmap Epigenomics Project to evaluate whether the lead variants at each locus reside in enhancer regions for tissue types of interest by comparing CHIP-seq signals for chromatin state markers in selected tissues with possible conceptual connections to back pain via roles involving chondrogenesis, vertebral development, muscle, and the central nervous system.^21,22^

Because two CBP-associated variants were found to be associated with height in prior published GWAS, we conducted *post hoc* region-specific secondary GWAS analyses accounting for height, among UKB participants from the discovery stage. Model 1 (CBP ~ SNP + age + sex + array + PC1 + … + PC10) examined associations between SNPs and CBP using logistic regression, among UKB participants from the interim data release. Model 2 (CBP ~ SNP + age + sex + + height+ array + PC1 + … + PC10) used similar methods, but also adjusted for height. Models 1 and 2 were compared descriptively with respect to the lead SNPs in *SOX5* and *CCDC26/GSDMC* that were identified in the discovery stage meta-analysis*,* to assess whether these SNP-CBP associations differed when including height as a covariate. Model 3 (CBP ~ SNP + age + sex + array + PC1 + … + PC10) was the same as Model 1, but analysis was conditional on the top height-associated variant in the region. Models 1 and 3 were compared descriptively with respect to the lead SNPs from region-specific analyses among participants from the UKB interim data release*,* to assess whether results for the lead SNPs differed when conditional on the top height-associated variant in the region. Model 4 was a linear regression model with additive genetic effects to examine for associations between each variant and height (height ~ SNP + age + sex + array + PC1 + … + PC10). Model 5 was the same as Model 4 (height ~ SNP + age + sex + array + PC1 + … + PC10), but analysis was conditional on the top CBP-associated variant in the region. Models 4 and 5 were compared descriptively with respect to the lead SNPs from region-specific analyses among participants from the UKB interim data release*,* to assess whether results for the lead SNPs differed when conditional on the top CBP-associated variant in the region (Supplemental Table S7).

We performed a two-sample MR to examine potential causal effects of height on CBP using significant variants associated with standing height from the GIANT consortium as the exposure, and the discovery phase meta-analysis of CBP, using R package MRbase.^23,24^ We used GWAS summary statistics for height described in Wood et.al^25^ and available through MR-Base for extraction of significant independent SNPs. We used the default MR-Base parameters for clumping (p-value≤5 x 10^-8^, r^2^ cut-off=0.001, clumping distance cut-off=10000kb). The European samples from 1000 Genomes were used to estimate LD between SNPs. We used only those SNPs from GIANT that overlapped with SNPs from our GWAS of CBP, and those with MAF>5%. In total we extracted 326 genetic instrumental variables. We conducted MR analyses using the inverse-variance weighted regression (IVW) approach as our primary analysis method, but also conducted additional analyses using other MR methods (MR-Egger regression, weighted median function, and weighted mode); presenting the results yielded from different MR methods is recommended to demonstrate sensitivity to different patterns of assumption violations.^23,24^ We used heterogeneity statistics from the IVW approach and forest plots to examine heterogeneity of exposure-outcome associations among different SNPs, examined violations of MR assumptions using funnel plots, and used the MR-Egger intercept test for directional horizontal pleiotropy. Results of the two-sample MR using all 326 SNPs are available in the S2-3 Appendices. Subsequently, we identified SNP outliers with potentially pleiotropic effects on CBP. We considered a SNP to have a potential pleiotropic effect on CBP if it had a p-value <0.05 using the Mendelian randomization pleiotropy residual sum and outlier test (MR-PRESSO^26^), or if it was associated with CBP (p<0.01) in the discovery phase meta-analysis. Eight SNPs were removed due to p<0.05 with MR-PRESSO, and an additional 6 SNPs were removed due to associations with CBP (p<0.01) (S2 Appendix). After exclusion of these 14 SNPs, the two-sample MR was repeated; these results using 312 SNPs are available in the S2-3 Appendices.

Given that 30% of UKB participants are related to at least one other person in the cohort, we also conducted post hoc secondary GWAS in UKB1 to examine whether relatedness might have influenced our results. These analyses used linear mixed-effect models (BOLT-LMM), adjusting for age, sex, study-specific covariates, and principal components. The statistical significance of GWAS results for UKB1 using BOLT-LMM were descriptively compared with the original results using logistic regression, for the lead variants achieving suggestive significance in the GWAS meta-analysis.

Finally, we used LDsr of summary-level GWAS results from the discovery stage to estimate heritability due to common autosomal SNPs and genetic correlations.^15^ We transformed the observed SNP heritability to the liability scale, in order to make heritability estimates for CBP comparable with traditional heritability estimates from twin studies.^27^ We used stratified LDsr to partition heritability across functional categories of the genome, using methods described previously by Finucane et al.^28^ The threshold for determining the statistical significance of 53 functional categories in partitioning heritability was set at p<9.4 x 10-4 (0.05/53). Further details of these methods are presented in the S3 Appendix. We used cross-trait LDsr and publicly available meta-GWAS results from LDhub to examine genetic correlations with a limited list of traits with strong conceptual links to CBP, including anthropometrics (standing height, waist circumference,hip circumference, BMI, and overweight/obesity), depression and depressive symptoms, osteoarthritis and rheumatoid arthritis.^29,30^

1. Fried LP, Borhani NO, Enright P, et al. The Cardiovascular Health Study: design and rationale. Ann Epidemiol 1991;1:263-76.

2. Dawber TR, Meadors GF, Moore FE, Jr. Epidemiological approaches to heart disease: the Framingham Study. Am J Public Health Nations Health 1951;41:279-81.

3. Suri P, Katz JN, Rainville J, Kalichman L, Guermazi A, Hunter DJ. Vascular disease is associated with facet joint osteoarthritis. Osteoarthritis Cartilage 2010;18:1127-32.

4. Smith BH, Campbell A, Linksted P, et al. Cohort Profile: Generation Scotland: Scottish Family Health Study (GS:SFHS). The study, its participants and their potential for genetic research on health and illness. Int J Epidemiol 2013;42:689-700.

5. Jordan JM. An Ongoing Assessment of Osteoarthritis in African Americans and Caucasians in North Carolina: The Johnston County Osteoarthritis Project. Trans Am Clin Climatol Assoc 2015;126:77-86.

6. Kherad M, Rosengren BE, Hasserius R, et al. Low clinical relevance of a prevalent vertebral fracture in elderly men--the MrOs Sweden study. The spine journal : official journal of the North American Spine Society 2015;15:281-9.

7. Orwoll E, Blank JB, Barrett-Connor E, et al. Design and baseline characteristics of the osteoporotic fractures in men (MrOS) study--a large observational study of the determinants of fracture in older men. Contemp Clin Trials 2005;26:569-85.

8. Lester G. The Osteoarthritis Initiative: A NIH Public-Private Partnership. HSS J 2012;8:62-3.

9. Hofman A, Brusselle GG, Darwish Murad S, et al. The Rotterdam Study: 2016 objectives and design update. Eur J Epidemiol 2015;30:661-708.

10. The Study of Osteoporotic Fractures (SOF): A Brief Introduction. (Accessed 10/07/2017, at <https://sofonline.epi-ucsf.org/docs/Study_Overview.pdf>.)

11. Spector TD, Williams FM. The UK Adult Twin Registry (TwinsUK). Twin Res Hum Genet 2006;9:899-906.

12. Sudlow C, Gallacher J, Allen N, et al. UK biobank: an open access resource for identifying the causes of a wide range of complex diseases of middle and old age. PLoS Med 2015;12:e1001779.

13. UK Biobank. Genotype imputation and genetic association studies of UK Biobank Interim Data Release, May 20152015.

14. Winkler TW, Day FR, Croteau-Chonka DC, et al. Quality control and conduct of genome-wide association meta-analyses. Nat Protoc 2014;9:1192-212.

15. Bulik-Sullivan BK, Loh PR, Finucane HK, et al. LD Score regression distinguishes confounding from polygenicity in genome-wide association studies. Nat Genet 2015;47:291-5.

16. Higgins JP, Thompson SG. Quantifying heterogeneity in a meta-analysis. Stat Med 2002;21:1539-58.

17. Kircher M, Witten DM, Jain P, O'Roak BJ, Cooper GM, Shendure J. A general framework for estimating the relative pathogenicity of human genetic variants. Nat Genet 2014;46:310-5.

18. Boyle AP, Hong EL, Hariharan M, et al. Annotation of functional variation in personal genomes using RegulomeDB. Genome Res 2012;22:1790-7.

19. GTEx Consortium. Human genomics. The Genotype-Tissue Expression (GTEx) pilot analysis: multitissue gene regulation in humans. Science 2015;348:648-60.

20. Watanabe K, Taskesen E, van Bochoven A, Posthuma D. Functional mapping and annotation of genetic associations with FUMA. Nat Commun 2017;8:1826.

21. Ernst J, Kellis M. ChromHMM: automating chromatin-state discovery and characterization. Nat Methods 2012;9:215-6.

22. Roadmap Epigenomics C, Kundaje A, Meuleman W, et al. Integrative analysis of 111 reference human epigenomes. Nature 2015;518:317-30.

23. Hemani G, Zheng J, Wade KH, et al. MR-Base: a platform for systematic causal inference across the phenome using billions of genetic associations. bioRxiv 2016.

24. Hemani G, Zheng J, Elsworth B, et al. The MR-Base platform supports systematic causal inference across the human phenome. Elife 2018;7.

25. Wood AR, Esko T, Yang J, et al. Defining the role of common variation in the genomic and biological architecture of adult human height. Nat Genet 2014;46:1173-86.

26. Verbanck M, Chen CY, Neale B, Do R. Detection of widespread horizontal pleiotropy in causal relationships inferred from Mendelian randomization between complex traits and diseases. Nat Genet 2018;50:693-8.

27. Zaitlen N, Kraft P. Heritability in the genome-wide association era. Hum Genet 2012;131:1655-64.

28. Finucane HK, Bulik-Sullivan B, Gusev A, et al. Partitioning heritability by functional annotation using genome-wide association summary statistics. Nat Genet 2015;47:1228-35.

29. Bulik-Sullivan B, Finucane HK, Anttila V, et al. An atlas of genetic correlations across human diseases and traits. Nat Genet 2015;47:1236-41.

30. Zheng J, Erzurumluoglu AM, Elsworth BL, et al. LD Hub: a centralized database and web interface to perform LD score regression that maximizes the potential of summary level GWAS data for SNP heritability and genetic correlation analysis. Bioinformatics 2017;33:272-9.
