# Supplementary material for: Genome-wide meta-analysis of 158,000 individuals of European ancestry identifies three loci associated with chronic back pain
Source: PLoS Genet. 2018 Sep 27;14(9):e1007601. doi: 10.1371/journal.pgen.1007601 (PMC6159857; doi:10.1371/journal.pgen.1007601)
Supplement: S2 Text — (DOCX) [file pgen.1007601.s021.docx]

This meta-analysis was approved by the Research and Development Committee VA Puget Sound Health Care System (RDIS 0010, MIRB 00903). Written or electronic consent was provided for all studies.

**Cardiovascular Heart Study**

University of Washington IRB Committee (00000109)

**Framingham Heart Study**

Boston University Medical Center (H-31304)

**Generation Scotland**

NHS Tayside Committee on Medical Research Ethics (05/S1401/89)

**Johnston County Osteoarthritis Project**

University of North Carolina at Chapel Hill/Centers for Disease Control and Prevention (92-0583)

**Mr. Os-Sweden**

Ethics Committee in Gothenburg (Gbd M 014-01)

Malmo - evalauted by the Ethics Committee in Lund (LU 693-00)

**Mr. Os-US**

Human Research Protection Program at the University of Minnesota (9804M00050)

Stanford University Administrative Panel on Human Subjects in Medical Research (13464 SQL 76147)

University of Pittsburgh Institutional Review Board (IRB980305)

Oregon Health & Science University (IRB #754)

University of California, San Diego Human Research Protections Program (071795)

California Pacific Medical Center Institutional Review Board (#23.103EXP)

University of California, San Francisco Human Research Protection Program (#H6613-15013-12A)

**Osteoarthritis Initiative**

Committee on Human Research at the University of California, San Francisco (10-00532)

**Rotterdam Study**

Medical Ethics Committee of the Erasmus Medical Center

**Study of Osteoporotic Fractures:**

University of Maryland, Baltimore Institutional Review Board (HM-HP-00040708-1)

Human Research Protection Program at the University of Minnesota (9104M03716)

University of Pittsburgh Institutional Review Board (IRB011218)

California Pacific Medical Center Institutional Review Board (#23.104EXP)

University of California, San Francisco Human Research Protection Program ( #11-06401)

**10,001 Dalmatians**

Ethical Board of the Medical School, University of Split, Croatia (003-08/11-03/0005)

Lothian NHS Board, South East Scotland Research Ethics Committees (11/AL/0222)

**TwinsUK:**

Westminster Research Ethics Committee (EC04/015)

**UK Biobank:**

UK Biobank Research Ethics Committee (11/NW/0382) (electronic consent)
