# Supplementary material for: Genome-wide meta-analysis of 158,000 individuals of European ancestry identifies three loci associated with chronic back pain
Source: PLoS Genet. 2018 Sep 27;14(9):e1007601. doi: 10.1371/journal.pgen.1007601 (PMC6159857; doi:10.1371/journal.pgen.1007601)

**Results from two sample MR for height as exposure and BP as outcome**

Table A3-1. Results from two sample MR for 326 and 312 SNPs.

| Method | 326 instruments | | | 312 instruments | | |
| --- | --- | --- | --- | --- | --- | --- |
|  | b | se | pval | b | se | pval |
| Inverse variance weighted | 0.098 | 0.023 | 4.2E-05 | 0.088 | 0.021 | 3.0E-05 |
| MR Egger | 0.088 | 0.067 | 0.19 | 0.086 | 0.060 | 0.15 |
| Weighted median | 0.127 | 0.032 | 5.3E-05 | 0.127 | 0.033 | 0.0001 |
| Weighted mode | 0.156 | 0.064 | 0.015 | 0.162 | 0.074 | 0.028 |

Table A3-2. Results of heterogeneity test.

| Number of instruments | Q | Q_df | Q_pval | I^2^ |
| --- | --- | --- | --- | --- |
| 326 | 499.35 | 325 | <1e-22 | 0.35 |
| 312 | 355.33 | 311 | 0.04 | 0.12 |

Table A3-3. Results of test for directional horizontal pleiotropy.

| Number of instruments | egger_intercept | se | pval |
| --- | --- | --- | --- |
| 326 | 3.1E-04 | 0.002 | 8.8E-01 |
| 312 | 9.0E-05 | 0.002 | 9.6E-01 |

Figure A3-1. Forest plots of single SNP MR before (A) and after (B) removing outliers.


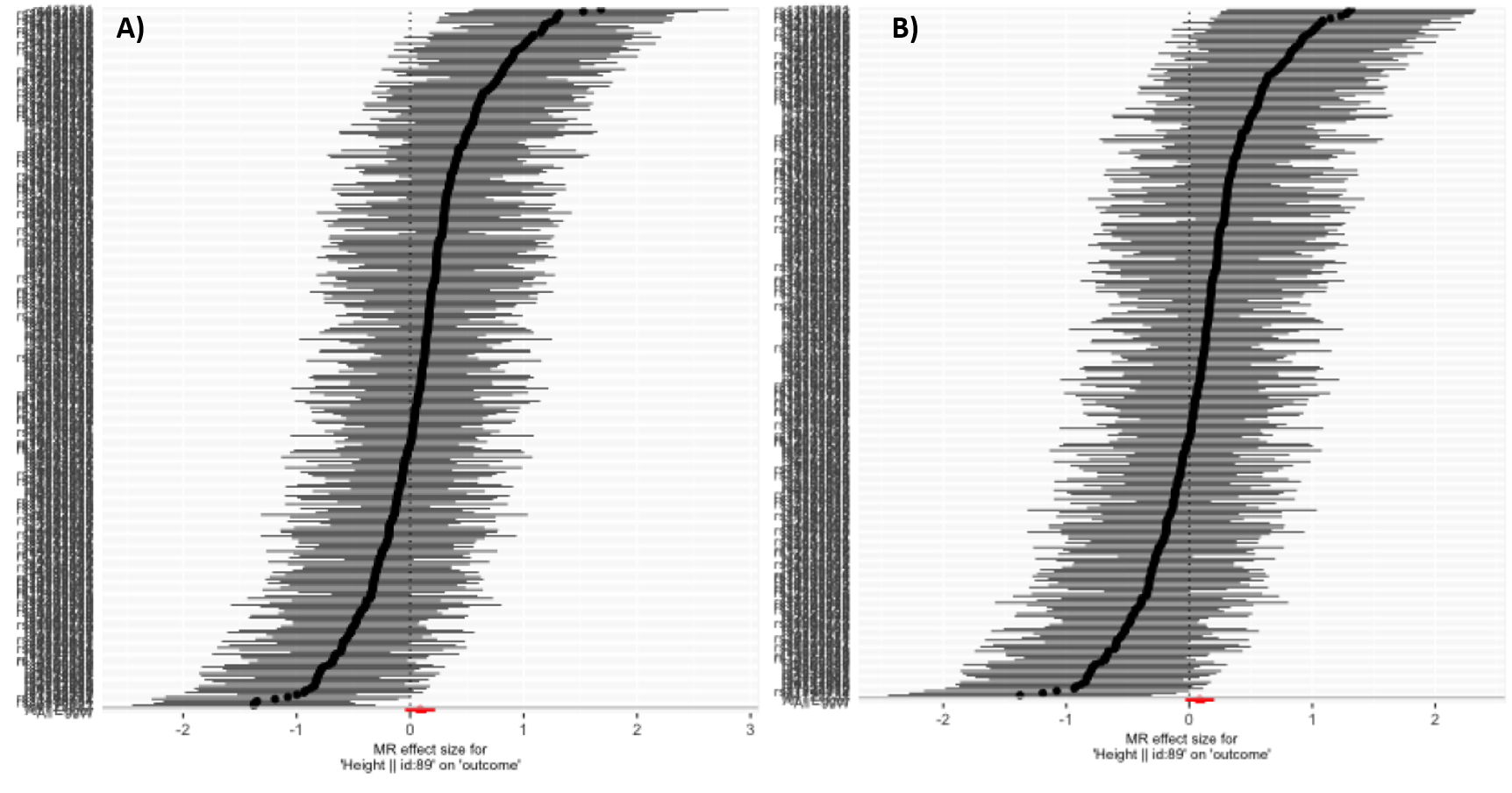


Figures A3-2. Funnel plots before (A) and after (B) removing outliers.


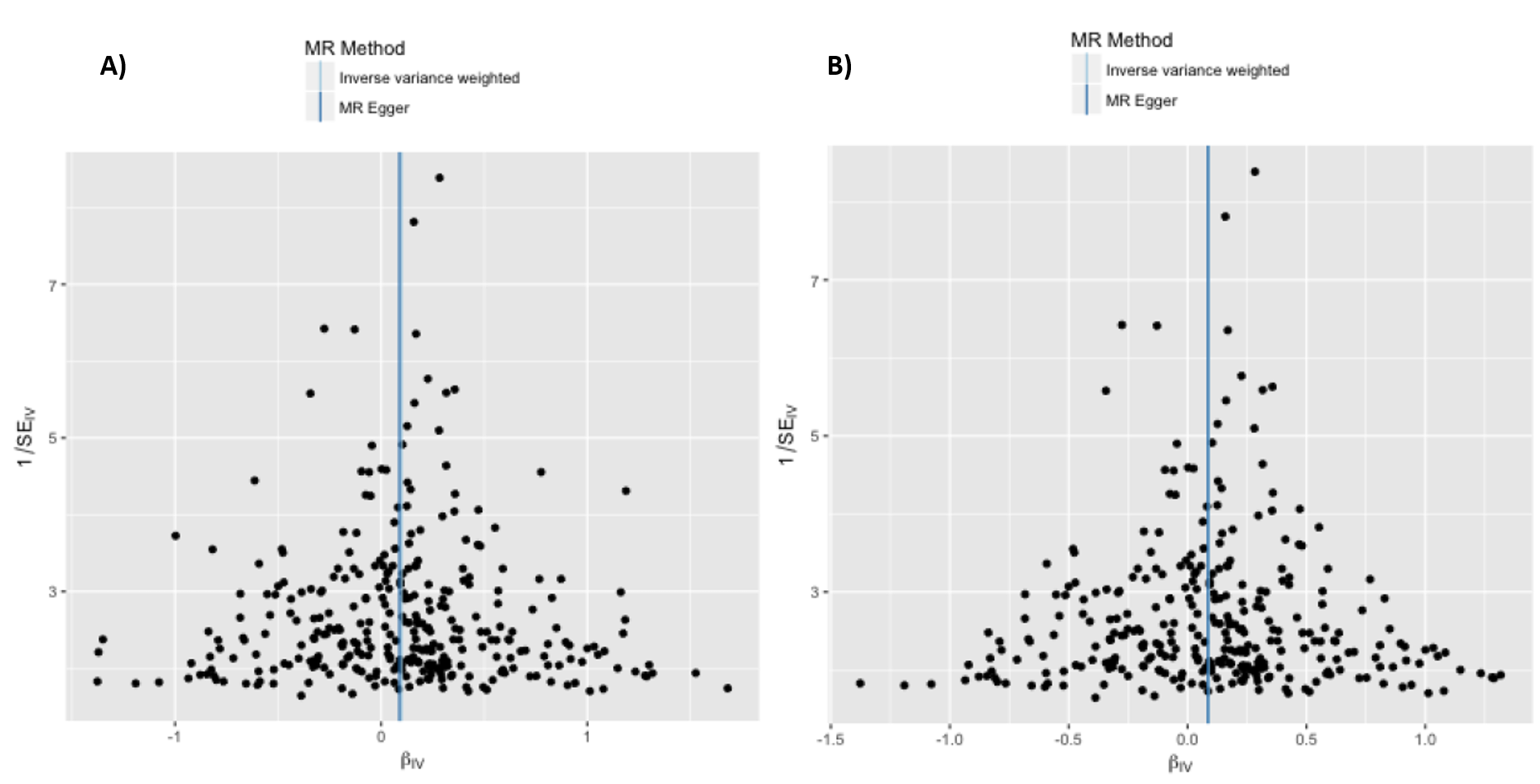


Figures A3-3. Leave-one-out sensitivity analysis before (A) and after (B) removing outliers.


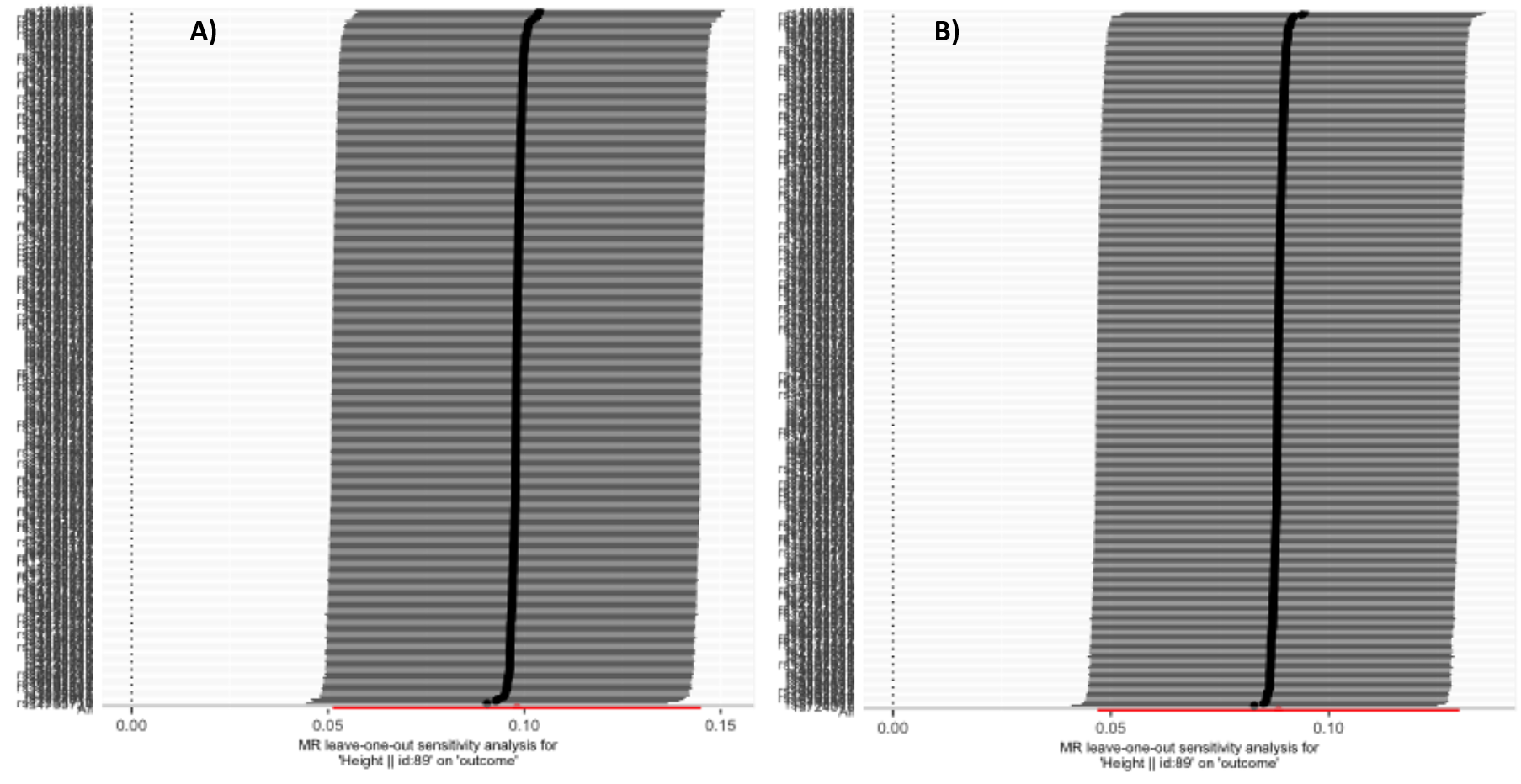

Supplement: S3 Appendix — (DOCX) [file pgen.1007601.s024.docx]
